# RT206 Couples Partial PPARα/γ Agonism with Ligand-Dependent Allosteric Potentiation of FXR Signaling in Liver Cells

**DOI:** 10.3390/biom16091316

**Published:** 2026-09-10

**Authors:** Manuela Leo, Carmen Cerchia, Antonio Laghezza, Francesca Rinaldi, Enrica Calleri, Vittorio Colantuoni, Fulvio Loiodice, Lina Sabatino, Antonio Lavecchia

**Affiliations:** 1 Department of Sciences and Technologies, University of Sannio, 82100 Benevento, Italy; 2 Drug Discovery Laboratory, Department of Pharmacy, University of Naples “Federico II”, 80131 Naples, Italy; carmen.cerchia@unina.it; 3 Department of Pharmacy-Drug Science, University of Bari “Aldo Moro”, 70126 Bari, Italy; antonio.laghezza@uniba.it (A.L.);; 4 Department of Drug Sciences, University of Pavia, 27100 Pavia, Italy; francesca.rinaldi@unipv.it (F.R.);

**Keywords:** PPARs, FXR, positive allosteric modulation, molecular docking and dynamics, gene expression profiling, steatosis, MASLD

## Abstract

Metabolic dysfunction-associated steatotic liver disease (MASLD) is a chronic disorder characterized by dysregulated lipid handling and bile acid signaling, processes controlled by PPARs and FXR. We report that RT206, a 2-aryloxy-3-phenyl-propanoic acid derivative, combines partial PPARα/γ agonism with positive allosteric modulation of FXR. Transactivation assays and endogenous gene-expression profiling confirmed activation of PPARα/γ-responsive genes. RT206 showed no intrinsic FXR agonism, yet it robustly potentiated FXR-dependent gene transcription in HepG2 cells in the presence of structurally distinct orthosteric agonists. Grating-coupled interferometry revealed weak interaction with apo-FXR that was enhanced by orthosteric ligands, consistent with cooperative ternary complex formation. Docking and molecular dynamics supported a model in which RT206 engages the non-orthosteric FXR S2 site, while orthosteric agonists occupy the S1 primary pocket. Comparison with guggulsterone revealed distinct predicted S2 interaction patterns associated with divergent transcriptional outcomes in vitro. In fatty acid-loaded HepG2 cells, RT206 combined with FXR agonists modulated FXR- and PPAR-regulated metabolic genes and modestly reduced neutral-lipid accumulation. These findings identify RT206 as a proof-of-principle scaffold that integrates partial PPARα/γ agonism with ligand-dependent potentiation of FXR activity and support its further evaluation in more advanced liver models.

## 1. Introduction

Metabolic dysfunction-associated steatotic liver disease (MASLD) is a highly prevalent chronic liver disease, affecting approximately 30% of adults worldwide [1,2]. MASLD encompasses a spectrum that begins with hepatic steatosis, arising primarily from the increased flux of free fatty acids released by insulin-resistant adipose tissue and, to a lesser but disproportionately elevated extent, from hepatic de novo lipogenesis [3]. This metabolic overload promotes lipotoxic stress and inflammatory signaling, including NF-κB-related chemokine and cytokine pathways, with activation of Kupffer cells and hepatic stellate cells, thereby sustaining chronic low-grade inflammation [4,5,6]. In a subset of patients, the disease progresses to metabolic dysfunction-associated steatohepatitis (MASH), characterized by hepatocellular injury, inflammation, and fibrogenesis, with major implications for morbidity and mortality [4,6]. The MASLD/MASH nomenclature was established in 2023 by a multisociety Delphi consensus, replacing the former NAFLD/NASH terminology; the current definition requires documented hepatic steatosis together with at least one cardiometabolic risk factor [7,8].

Metabolic stress, inflammatory signaling, and endocrine and immune inputs are deemed to contribute to the MASLD “multiple-hit” pathogenesis, acting in parallel and feed-forward loops, rather than as a single linear cascade [5,6].

Since metabolic alterations and inflammation are implicated in MASLD development and progression, ligand-activated nuclear receptors have attracted considerable attention as integrative metabolic sensors and transcriptional regulators [9,10,11].

Farnesoid X receptor (FXR), also known as the bile acid receptor, is highly expressed in the liver and plays a central role in bile acid homeostasis. Upon activation, FXR limits bile acid synthesis by inducing the small heterodimer partner (SHP), which contributes to repression of *CYP7A1*, the gene encoding the rate-limiting enzyme in the pathway, and promotes bile acid export through transporters including *BSEP* and *OSTα*/*OSTβ* [12]. FXR is also expressed in the intestine, where it coordinates enterohepatic feedback signaling and contributes to systemic metabolic control. In addition, FXR exerts pleiotropic anti-inflammatory effects through several overlapping mechanisms [13,14].

Recent regulatory approvals of resmetirom and semaglutide for selected adults with noncirrhotic MASH and moderate-to-advanced fibrosis have changed the therapeutic landscape [15,16]. Nevertheless, substantial unmet need remains for complementary strategies capable of modulating interconnected lipid and bile acid pathways with acceptable long-term tolerability. FXR-directed drug development has included obeticholic acid (OCA), which improved liver histology in patients with MASH but was associated with dose-dependent pruritus and adverse lipid changes [17]. These findings support the therapeutic relevance of FXR engagement while highlighting tolerability limitations that motivate alternative strategies to modulate FXR signaling more selectively [17].

Peroxisome proliferator-activated receptors (PPARs) have also emerged as appealing targets for MASH, as their modulation simultaneously addresses several pathological features of the disease. PPARs comprise three subtypes (α, β/δ, and γ) with distinct tissue distributions and roles in energy, glucose, and lipid metabolism [18,19]. However, the clinical use of PPARα or γ agonists has been limited by partial efficacy or safety concerns. More recently, selected dual or pan-agonists, such as lanifibranor, have shown promising results in clinical studies in MASH, with Phase 3 evaluation ongoing [20,21]. These data support increasing interest in partial agonists or modulators that fine-tune receptor activity rather than fully activate canonical programs, potentially reducing adverse effects associated with full agonism [22].

In this context, we previously described a series of 2-aryloxy-3-phenyl-propanoic acids and identified selected compounds as ligands with enhanced potency toward PPARα while maintaining comparable potency and partial agonism on PPARγ [23]. Building on this series, we selected compound **15** (RT206) as a balanced partial PPARα/γ agonist and investigated its receptor pharmacology. In hepatoma cells, RT206 activated canonical PPAR targets and, unexpectedly, potentiated FXR-dependent transcription in the presence of orthosteric FXR agonists. Grating-coupled interferometry (GCI) and computational analyses supported RT206 binding to a non-orthosteric FXR site consistent with positive allosteric modulation. We further evaluated this mechanism in a steatotic HepG2 model to determine whether cooperative FXR potentiation was preserved under lipid-loading conditions. These findings provide a chemical-biology framework for targeting non-orthosteric nuclear receptor sites to modulate transcriptional networks relevant to chronic liver disease.

## 2. Materials and Methods

### 2.1. Chemicals and Reagents

Rosiglitazone (PPARγ agonist, #71740) was purchased from Cayman (Ann Arbor, MI, USA); WY-14643 (PPARα agonist, #HY-16995) from MedChem Express (Monmouth Junction, NJ, USA); GW4064 (FXR agonist, #S2782) from Selleckchem (Houston, TX, USA); obeticholic acid (OCA, FXR agonist, #SML3096) and (*Z*)-Guggulsterone (GG, FXR antagonist, #G5168) from Merck (Milan, Italy). All of them were dissolved in DMSO. RT206 was synthesized as reported previously [23].

### 2.2. Cell Culture and Treatments

HepG2 (ATCC HB-8065) and HEK293T (ATCC CRL-3216) cells were purchased from the American Type Culture Collection (ATCC, Manassas, VA, USA). HepG2 cells were cultured in DMEM or MEM supplemented with 10% *v*/*v* heat-inactivated fetal bovine serum, 0.1 mM NEAA, and 100 U mL^−1^ penicillin/streptomycin and incubated at 37 °C in a humidified incubator with 5% CO_2_ under standard growth conditions. HEK293T cells were grown in DMEM high glucose, supplemented with sodium pyruvate, 100 U mL^−1^ penicillin/streptomycin and 10% fetal bovine serum (FBS) at 37 °C and 5% CO_2_. Cells were routinely tested for mycoplasma contamination and cultured for no more than 12 passages.

HepG2 cells (8 × 10^5^) were seeded in 6-well plates in complete medium and left overnight in the incubator to enhance adherence. The next day, the medium was replaced with a serum-free medium, and the cells were exposed to GW4064 (1 µM), OCA (10 µM), WY-14643 (10 µM), and Rosiglitazone (1 µM), alone or in combination with RT206 (5, 10, 20 µM) for 24 h. Cells were also treated with GG (5, 10, 20, 30, and 40 µM) alone or in combination with GW4064 (1 µM) for 24 h. As a control for each experiment, cells were exposed to a medium containing DMSO (0.1%), used as a vehicle to dissolve the compounds employed. All treatments and control experiments were performed in serum-free medium.

### 2.3. Transactivation Assay

HepG2 cells (5 × 10^4^) were seeded in 96-well plates, and transfection was performed using EndoFectin™ HepG2 (#EF006, GeneCopoeia, Rockville, MD, USA), according to the manufacturer’s instructions. Each well received 20 ng of GAL4–NR-LBD or full-length FXR plasmid, 40 ng of reporter plasmid, and 40 ng of pCMVβgal. Twenty-four hours after transfection, the medium was replaced with complete MEM without FBS, containing either 0.1% DMSO or the indicated concentrations of ligands (WY-14643, Rosiglitazone, GW4064, RT206 or GG), in triplicate, for 20 h. Cells were then lysed and analyzed for luciferase and β-galactosidase activities using a VICTOR3 V Multilabel Plate Reader (PerkinElmer, Waltham, MA, USA). Luciferase activity was normalized to β-galactosidase activity and expressed as relative activity compared with the response to the standard ligands WY-14643 (PPARα), Rosiglitazone (PPARγ), or GW4064 (FXR), which were set to 100%.

HEK293T cells (3 × 10^4^) were seeded in 96-well plates, the medium was changed to Opti-MEM without supplements, and cells were transiently transfected with one pFA-CMV-hNR-LBD clone, pFR-Luc, and pRL-SV40 using Lipofectamine LTX reagent (Invitrogen, Carlsbad, CA, USA), according to the manufacturer’s instructions. Five hours after transfection, cells were incubated with the test compounds in Opti-MEM supplemented with penicillin (100 U/mL), streptomycin (100 μg/mL) and 0.1% DMSO. Transfections were carried out as previously reported [24].

### 2.4. RNA Extraction, cDNA Synthesis and qPCR Analysis

Total RNA was extracted from cells in culture using TRIzol™ Reagent (15596018, Invitrogen, Carlsbad, CA, USA) according to the manufacturer’s instructions. cDNA synthesis was performed using the SuperScript™ II First-Strand Synthesis System (18064014, Invitrogen). Quantitative real-time PCR (RT-qPCR) was carried out using PowerUp™ SYBR™ Green Master Mix (A25741, Applied Biosystems, Foster City, CA, USA) and acquired by QuantStudio5. Relative gene expression levels were calculated using the 2^−ΔΔCt^ method [25], where ΔCt = Ct_target_ − Ct_18S_ and ΔΔCt = ΔCt_treated_ − ΔCt_control_. The nucleotide sequences of the primers used for RT-qPCR analysis are reported in Table S5.

### 2.5. Protein Extraction and Western Blotting Analysis

At the end of each treatment, cells were harvested by trypsinization and lysed in an ice-cold lysis buffer containing 25 mM Tris-HCl, pH 7.5, 150 mM NaCl, 2 mM EDTA, 1% Triton X-100, 1% sodium deoxycholate, 0.1% SDS, and a cocktail of protease and phosphatase inhibitors. After heating at 95 °C for 5 min, 30 µg of protein extracted from each sample was loaded onto non-reducing polyacrylamide gels, transferred onto PVDF membranes, and blocked with 5% non-fat dry milk for 1 h at room temperature. Subsequently, the membranes were incubated with primary antibodies overnight at 4 °C. Antibodies to PPARγ (#2443) were purchased from Cell Signaling Technologies (Danvers, MA, USA); PPARα (#Ab227074) from Abcam (Cambridge, UK); and α-Tubulin (#T5168) from Merck (Table S6). Membranes were washed and incubated with secondary antibodies for 1 h at room temperature. Anti-rabbit and anti-mouse antibodies conjugated to HRP were purchased from Cell Signaling Technologies. After washing, bands were detected by Clarity Western ECL Substrate (#1705062 or #1705061, BIORAD, Hercules, CA, USA) using the ChemiDoc apparatus. Bands were quantified using the ImageLab software, version 6.1.0 [26].

### 2.6. Cell Viability Assay

HepG2 cells (5 × 10^4^) were seeded in 96-well plates in complete medium and left overnight in the incubator to enhance adherence. The next day, the medium was replaced, and the cells were exposed to RT206 (5, 10, 20, 40, 60, 80 and 100 µM), GW4064 (1 µM), OCA (10 µM), WY-14643 (10 µM), Rosiglitazone (1 µM), alone or in combination with RT206 for 24 h. Cells were also treated with GG (5, 10, 20, 30, and 40 µM) alone or in combination with GW4064 (1 µM) for 24 h. Viable cells were measured with Prestoblue™ Cell Viability Reagent (#A13262, Invitrogen) and the fluorescence intensity (FI) was detected with a Tecan Infinite-Pro 200 plate reader (TECAN, Mannedorf, Switzerland) using excitation at 540 nm and emission at 595 nm [25].

### 2.7. Grating Coupled Interferometry (GCI) Experiments

The recombinant human FXR ligand-binding domain (FXR-LBD) used in the GCI experiments was custom-produced in a bacterial expression system and purified under contract by Biogem S.c.ar.l. (Ariano Irpino, Italy). The protein was used as supplied without further purification.

GCI analyses were performed on a Creoptix WAVE instrument (Malvern Panalytical, Wädenswil, Switzerland) controlled by the Creoptix WAVE control software (version 4.5.18). For the experiments, a 4PCH sensor chip functionalized with a high-capacity polycarboxylate layer was used. The composition of the running buffer was 10 mM HEPES, pH 7.4, 150 mM NaCl (with 1% dimethyl sulfoxide, DMSO, for kinetic and screening analyses). The chip temperature was maintained at 25 °C. FXR was immobilized on the 4PCH sensor chip by amine coupling. Two channels were activated by the injection of a mixture of 200 mM N-(3-dimethylaminopropyl)-N’-ethylcarbodiimide hydrochloride (EDC) and 50 mM N-hydroxysuccinimide (NHS) for 420 s at a flow rate of 10 µL/min. Then, two injections of a 100 µg/mL FXR solution in 20 mM sodium acetate, pH 4.5, were carried out at 10 µL/min for 600 s each in the first channel, while the second was used as a reference. The chip unreacted groups were blocked by injecting 1 M ethanolamine, pH 8.5, for 300 s at 10 µL/min. Kinetic analyses were performed in a concentration range of 1.6–200 µM for RT206, 3.1–400 nM for GW4064, 39 nM–5 µM for OCA and 1.25–160 µM for GG. Eight concentrations were analyzed, selecting a dilution factor of 2, and 2 replicate analyses were carried out for each concentration. A flow rate of 50 μL/min was applied, with an association time of 120 s and a dissociation time of 180 s. A DMSO calibration curve (5 points) was built for each analysis in the concentration range 0.9–2.9% DMSO. Kinetic analysis of RT206 was also performed in the presence of a fixed concentration of GW4064 (1 μM) or OCA (10 or 20 μM) in the running buffer.

The experiments to distinguish allosteric binders from competitive binders were carried out using the screen modality by injecting different concentrations of orthosteric ligands individually or in mixture with RT206. GW4064 was analyzed at concentrations of 50 nM, 500 nM, 1 μM, 5 μM and 10 μM, with RT206 at 500 nM, 1 μM, 5 μM, 10 μM, 20 μM, 50 μM and 100 μM. Concentrations of 1, 5, 10 and 20 μM were selected for OCA, with RT206 at 10, 20, 40, 80 and 100 μM. GG was also analyzed in combination with GW4064, using concentrations of 5 and 10 µM for GW4064 and 100 µM for GG. Analyses in the screen modality were carried out at a fixed flow rate of 50 μL/min, using the same association and dissociation times as the kinetic analyses. Triplicate analyses were performed for each sample. Also in this case, a 5-point calibration curve was built for DMSO in the concentration range 0.9–2.9%.

### 2.8. Computational Chemistry

#### 2.8.1. Protein and Ligand Preparation

The following crystal structures were selected as starting models for the computational studies: PPARα in complex with fenofibric acid (PDB 7BQ0) [27], PPARγ in complex with ligand S-2 (PDB 3HOD) [23], and FXR structures including the apo form (PDB 6HL0) and complexes with CDCA (PDB 6HL1) [28] or GW4064 (PDB 3DCT) [29]. Protein structures were prepared using the Protein Preparation Workflow in Maestro (Protein Preparation Workflow; Epik, Schrödinger, LLC, New York, NY, USA, 2025; Impact, Schrödinger, LLC, New York, NY, USA; Prime, Schrödinger, LLC, New York, NY, USA, 2025). All crystallographic waters and non-essential heteroatoms were removed, hydrogens were added, and bond orders, formal charges, and atom types were assigned. Protonation states and tautomers of titratable residues were sampled, and the hydrogen-bonding network was optimized at physiological pH. Each structure was then subjected to restrained energy minimization with the Impref module using OPLS4, applying a 0.3 Å RMSD restraint on heavy atoms relative to the experimental coordinates. Unless otherwise stated, default parameters were used. Ligands were sketched in Maestro (2D Sketcher) and prepared with LigPrep (LigPrep, Schrödinger, LLC, New York, NY, USA, 2025) to generate 3D geometries, stereoisomers, and relevant tautomeric/protonation states at pH 7.0.

#### 2.8.2. Docking and MM-GBSA Rescoring

Molecular docking was performed with Glide (Glide, Schrödinger, LLC, New York, NY, USA, 2025). Docking grids were defined by centering a cubic box on the co-crystallized ligand of each receptor. Grid dimensions were set to 15 × 15 × 15 Å for PPARα and 10 × 10 × 10 Å for PPARγ. A van der Waals radii scaling factor of 0.8 was applied to receptor atoms, and ligands were treated as fully flexible. Docking was run using default Glide settings, without positional or interaction constraints. Binding poses were ranked using the GlideScore scoring function. The docking protocol was validated by re-docking the corresponding co-crystallized ligands into their binding sites, producing poses consistent with the experimental conformations (RMSD < 2.0 Å). RMSD values were calculated over ligand heavy atoms. Final poses for the compounds under study were selected based on GlideScore, overlap with known binding modes, and preservation of key interactions supported by experimental evidence. MM-GBSA calculations were carried out with Prime (Prime, Schrödinger, LLC, New York, NY, USA, 2025) on the selected docked complexes using predefined dielectric constants, the OPLS4 force field, and the VSGB solvation model [30]. Prime MM-GBSA default settings were used unless otherwise specified. More negative values (ΔG_bind) indicate stronger predicted binding. MM-GBSA rescoring was also used to compare ligands and stereoisomers. Structural figures were generated with PyMOL (The PyMOL Molecular Graphics System, Version 2.0; Schrödinger, LLC).

#### 2.8.3. FXR Binding-Site Identification with SiteMap

Potential ligand-binding sites in FXR were identified using SiteMap on the prepared FXR structures: apo FXR (PDB 6HL0), FXR bound to CDCA (PDB 6HL1), FXR bound to GW4064 (PDB 3DCT), and the rat FXR structure in complex with OCA (PDB 1OSV) [31]. SiteMap identifies and prioritizes putative ligand-binding pockets by evaluating a combination of geometric and physicochemical features, such as pocket volume, degree of enclosure/solvent exposure, hydrophobic–hydrophilic complementarity, and the strength of local van der Waals contacts. These descriptors are condensed into two composite indices, namely SiteScore and the Druggability Score (Dscore), which are benchmarked against a curated set of high-affinity (submicromolar) binding sites. Accordingly, SiteScore values above 1.0 and Dscore values close to or exceeding 1 are generally taken as indicative of cavities with favorable druggability. In the present analysis, only sites containing at least 15 site points were retained; calculations employed the standard site-point grid spacing (0.7 Å) and the more stringent hydrophobicity definition. Retained pockets were then ranked by SiteScore to prioritize the most plausible ligand-binding regions and filter out sites unlikely to support stable binding.

#### 2.8.4. FXR Docking and IFD

Following site identification, docking simulations of RT206 were performed to identify a binding pose in the S2 backdoor pocket of FXR in complex with GW4064 and CDCA. To maintain consistency with the human FXR structure across simulations and to match the experimental conditions used in this work, CDCA was manually modified to OCA. Docking of RT206 in the S1 site was carried out by centering the grid on the co-crystallized ligand in the 6HL1 structure. Because of the rigid steroidal scaffold, docking of GG into the S2 backdoor pocket, alone and in the presence of both orthosteric agonists (GW4064 and OCA), required the Induced Fit Docking (IFD) workflow (Induced Fit Docking protocol; Glide, Schrödinger, LLC, New York, NY, 2024; Prime, Schrödinger, LLC, New York, NY, USA, 2025) using the 6HL1 structure. In IFD, ligands are first docked with softened van der Waals potentials to generate candidate poses, after which Prime is used to predict and optimize side-chain conformations (and locally minimize residues near the ligand) to account for receptor flexibility. The ligand is then re-docked into the induced-fit receptor conformations using standard Glide settings, and final poses are ranked using an IFDScore that combines docking and protein-structure energetics.

#### 2.8.5. Molecular Dynamics (MD) Simulations and Analysis

The protein–ligand complexes obtained from docking or IFD were subjected to MD simulations using Desmond (Schrödinger Release 2025-4: Desmond Molecular Dynamics System, D. E. Shaw Research, New York, NY, USA, 2025; Maestro-Desmond Interoperability Tools, Schrödinger, New York, NY, USA, 2025). The simulations were performed retaining the coactivator peptide associated with each structure. Each system was solvated in an orthorhombic box with a 10 Å buffer using the TIP3P water model, neutralized by addition of counterions, and supplemented with 0.15 M NaCl to mimic physiological ionic strength. Simulations employed the OPLS4 force field [32]. Systems were relaxed using the standard Desmond relaxation protocol and then simulated for 1000 ns (1 μs) under the NPT ensemble. Temperature was maintained at 300 K using a Nose–Hoover thermostat, and pressure was maintained at 1 atm using the Martyna–Tobias–Klein barostat. For each system, three independent replicas were run using random seeds. Backbone Cα RMSD profiles for all replicas of each simulated system are reported in Figure S1.

Trajectory clustering was performed using Desmond Trajectory Clustering based on the RMSD matrix computed over a specified atom set (protein backbone), employing the affinity propagation algorithm. A frame extraction interval of 10 was used, and the maximum number of output clusters was set to 20. For each system, the representative structure from the most populated cluster was selected for subsequent analysis. Protein–ligand interaction patterns and persistence were evaluated using the Simulation Interaction Diagram tool. Finally, the representative structure was used for Prime MM-GBSA calculations. Additional trajectory processing and analysis were conducted using Python scripts provided by Schrödinger 2024-4.

### 2.9. Induction of the Steatosis Model

Steatosis was induced using a fatty acid (FA) mixture prepared according to a previously described protocol (https://www.protocols.io/view/preparation-of-bsa-complexed-free-fatty-acids-for-261genr6og47/v1 accessed on 1 January 2024) [33]. Sodium oleate (O) and sodium palmitate (P) were dissolved and mixed at a 2:1 molar ratio. The final FA concentration of the mixture was 0.5 mM; specifically, 2.2 µL O and 1.1 µL P were added per mL of solution. FAs were complexed with 10% fatty acid-free bovine serum albumin (BSA) at a 5:1 molar ratio (FFA: BSA). The final BSA concentration was 0.67%. A 50/50 (*v*/*v*) ethanol/H_2_O solution was used as a negative control. To induce the “standard” intracellular lipid accumulation, HepG2 cells were treated with the FA mixture alone or together with the compounds for 24 h (24 h model). Alternatively, the FA-containing medium was removed after 24 h and replaced with a serum-free medium containing either 0.1% DMSO or the investigated compounds for the subsequent 24 h (24 h + 24 h model).

### 2.10. Oil Red O Staining and Lipid Quantification

Oil Red O (ORO, #00625, Merck) stock solution was prepared according to the manufacturer’s instructions. After the treatments, cells were fixed with 10% formalin for 1 h at room temperature, washed with distilled water, incubated with 60% isopropanol for 5 min and stained for 10 min at room temperature with an ORO working solution freshly prepared by mixing 6 mL of ORO stock solution and 4 mL of distilled water. Following washing with distilled water, stained lipid droplets were visualized under bright-field microscopy. For quantitative analysis, plates were air-dried and lipids were eluted with 100% isopropanol for 10 min and collected, and absorbance was measured at 510 nm using a spectrophotometer. Then, for normalization, cells were incubated with Crystal violet (dissolved in 30% glacial acetic acid) for 10 min at room temperature and washed with distilled water. Plates were air-dried and incubated with 100% methanol for 10 min. The methanol phase was collected and absorbance measured at 570 nm using a spectrophotometer. The total lipid content was calculated as the ratio between ORO and Crystal violet absorbance.

### 2.11. Fluorescence Microscope and Flow Cytometry Analysis of BODIPY Staining

HepG2 cells (4 × 10^5^) were seeded in 6-well plates and allowed to adhere overnight. Following steatotic induction and/or treatments, cells were fixed with 4% PFA for 15 min, washed with PBS, permeabilized with 0.1% Triton-X-100, incubated with 1 µM BODIPY (#D3922, Invitrogen) for 15 min at 37 °C, washed with PBS, and observed under a fluorescence microscope. Nuclei were stained with DAPI. Fluorescence was captured at the same settings for each sample using a Nikon (Tokyo, Japan) microscope and the NIS-Elements software Ver 7.00.00. Alternatively, following steatotic induction and/or treatments, cells were washed with PBS and incubated with 1 µM BODIPY for 15 min at 37 °C. Cells were then harvested by trypsinization, centrifuged at 2000 rpm for 5 min, washed, and collected for flow cytometry analysis. Fluorescence intensity of lipid droplets was quantified as mean fluorescence intensity (MFI) in the FITC channel (Celesta flow cytometer, BD Biosciences, Franklin Lakes, NJ, USA). Data were acquired by the FACS Diva software, version 9.2 (BD) [34].

### 2.12. Statistical Analysis

Graphs were prepared and statistical analyses performed using GraphPad Prism 9. Unless otherwise stated, results are presented as mean ± standard deviation (SD). For experiments involving systematic comparisons among multiple groups, statistical significance was assessed by one-way analysis of variance (ANOVA) followed by Dunnett’s multiple-comparisons test or by two-way ANOVA followed by Tukey’s multiple-comparisons test, as appropriate. Unpaired Student’s *t*-tests were used for specific pre-specified pairwise comparisons between two experimental conditions. When Student’s *t*-test was used for multiple comparisons, Bonferroni correction was applied and indicated in the corresponding figure legends. A paired Student’s *t*-test was applied for GCI data analysis. Cell-based data are presented as mean ± SD, with the actual n-values of the experiments indicated in each figure legend. GCI kinetic measurements were performed using duplicate injections at each concentration, whereas mixture-screening experiments were performed in triplicate.

## 3. Results

### 3.1. Structure-Based Characterization of RT206 as a Balanced Partial PPAR Agonist

In a previous study, we characterized a series of synthetic compounds endowed with PPAR agonism [23], prioritizing molecules exhibiting efficacy below 100% on PPARα and PPARγ. Six candidates, *R*-**2**, *S*-**4**, **9**, **12**, **14**, and **15**, were further evaluated and ranked according to EC_50_ values. *S*-**4** and **15** emerged as the most potent compounds, displaying low micromolar activity (Table S1).

To refine the binding hypotheses, *S*-**4** and **15** were docked into PPARα and PPARγ ligand-binding domains (LBDs) and rescored using MM-GBSA. Compound **15** showed a consistent energetic advantage, driven by more favorable interaction energies and desolvation contributions. The preference for the *S*-enantiomer aligns with the stereochemical bias of *S*-configured ligands within the canonical Y-H-Y hydrogen-bonding network of the activation function-2 (AF-2) region on helix 12 (Table S2) [35].

**Figure 1:**
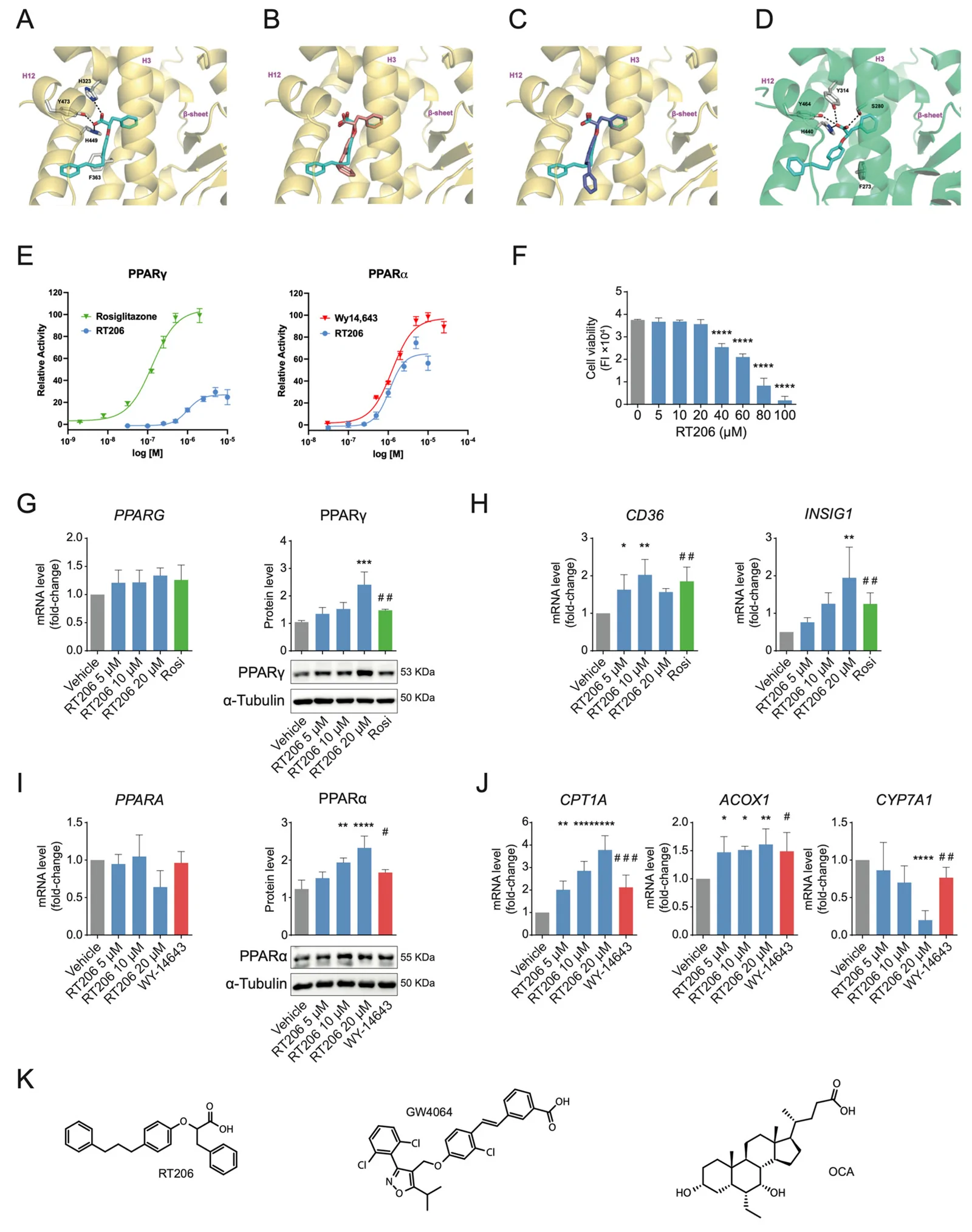
Predicted PPARα/γ binding modes and partial agonist activity of RT206 in hepatoma cells. Predicted binding mode of **15** (RT206) (cyan sticks) into (**A**) PPARγ and (**D**) PPARα LBDs, represented as yellow (PDB 3HOD) and green (PDB 7BQ0) ribbon models, respectively. Only amino acid residues discussed in the main text are displayed (white sticks) and labeled. H-bonds discussed in the text are depicted as dashed black lines. Overlay of **15** with *S*-**2** ((**B**), salmon sticks, PDB 3HOD) and *S*-**4** ((**C**), slate sticks, PDB 3HO0) co-crystallized into PPARγ LBD. (**E**) RT206 acts as a partial PPARα/γ agonist in GAL4-PPAR transactivation assays performed in HepG2 cells. The EC_50_ values were 1.10 ± 0.07 μM for PPARα and 1.02 ± 0.08 μM for PPARγ, with corresponding Emax values of 65% and 28%, respectively. (**F**) Assessment of HepG2 cell viability (n = 3) after exposure to the indicated concentrations of RT206 for 24 h. (**G**) Assessment of *PPARG* mRNA (n = 3) (left) and protein (n = 3) (right) levels in HepG2 cells treated for 24 h with the vehicle (DMSO), the indicated doses of RT206, or the PPARγ agonist Rosiglitazone (Rosi, 1 µM), as positive control. (**H**) Expression of *CD36* (n = 4) and *INSIG1* (n = 4), mRNAs of PPARγ target genes, in cells treated as in (**G**). (**I**) *PPARA* mRNA (left) (n = 4) and protein levels (right) (n = 4) in HepG2 cells treated for 24 h with the vehicle (DMSO), the indicated doses of RT206, or the PPARα agonist WY-14643 (10 µM), as positive control. (**J**) RT-qPCR analysis of *CPT1A* (n = 6), *ACOX1* (n = 4), and *CYP7A1* (n = 6) mRNAs, PPARα target genes, in HepG2 cells treated as in (**I**). (**K**) Chemical structures of RT206 and the FXR agonists GW4064 and obeticholic acid (OCA). All histograms show normalization of mRNA or protein levels to 18S or α-Tubulin in RT-qPCR and Western blot analysis, respectively. For panels (**F**–**J**), asterisks indicate comparisons of RT206 doses vs. vehicle by one-way ANOVA followed by Dunnett’s multiple-comparisons test: * *p* < 0.05, ** *p* < 0.01, *** *p* < 0.001, and **** *p* < 0.0001. Hash symbols indicate comparisons of the reference agonist vs. vehicle by an unpaired two-tailed Student’s *t*-test: # *p* < 0.05, ## *p* < 0.01 or ### *p* < 0.001.

Detailed analysis suggested that compound **15** adopts a binding orientation closely resembling that of its structural analogue, *S*-**2**, whose PPARγ co-crystal structure we previously reported [23]. In PPARγ, **15** is predicted to form a hydrogen-bonding network through its carboxylate group with Y473, H323, and H449, while its aromatic scaffold engages in π-stacking with F363, stabilizing the ligand within the pocket (Figure 1A). Superposition with co-crystallized *S*-**2** and *S*-**4** structures (Figure 1B,C) demonstrated the substantial overlap of both the acidic headgroup and aromatic core, consistent with the canonical binding mode of acidic PPARγ ligands that stabilize helix 12 and promote co-activator recruitment via the AF-2 surface [36].

In PPARα, **15** is predicted to adopt a similar orientation, with the carboxylate establishing hydrogen bonds with Y464, Y314, H440, and S280, and forming a π-stacking interaction with F273 (Figure 1D). Based on these structural and energetic considerations, compound **15** was selected for functional validation and is hereafter referred to as RT206.

### 3.2. RT206 Acts as a PPARα and PPARγ Ligand in Transactivation Assays and Hepatoma Cells

To validate the partial agonist profile of RT206, we performed GAL4-PPAR transactivation assays in HepG2 cells. Transient transfections yielded reproducible reporter activity consistent with our previously published data [23]. Dose–response curves after 20 h treatment (Figure 1E) showed EC_50_ values of 1.02 ± 0.08 μM for PPARγ (E_max_ 28%) and 1.10 ± 0.07 μM for PPARα (E_max_ 65%), confirming balanced partial agonism in a heterologous reporter system.

We next examined endogenous receptor activation in HepG2 cells. Cytotoxicity assessed by Prestoblue^TM^ revealed no significant reduction in viability up to 20–30 μM, whereas toxicity became evident at 40 μM (Figure 1F). Subsequent experiments were therefore conducted at 5, 10, and 20 μM for 24 h.

*PPARG* mRNA levels remained unchanged in RT-qPCR, whereas protein abundance increased dose-dependently, consistent with ligand-dependent stabilization and prolonged receptor half-life (Figure 1G) [37,38].

Consistently, the mRNA levels of *CD36* and *INSIG1*, two PPARγ target genes involved in lipid uptake and metabolic regulation, were induced by the treatment [39]. *INSIG1* showed a dose-dependent increase, whereas *CD36* reached its highest mean induction at 10 µM and showed a lower response at 20 µM, still above basal levels (Figure 1H). Although reporter assays indicated reduced intrinsic efficacy (partial E_max_), the magnitude of endogenous gene induction was comparable to that observed with the full agonist Rosiglitazone under these experimental conditions, a phenomenon that can reflect promoter context, chromatin environment, and co-regulator availability in native loci [40].

*PPARA* mRNA levels were also unchanged, whereas PPARα protein abundance increased dose-dependently (Figure 1I). The PPARα target genes *CPT1A* and *ACOX1*, encoding key enzymes of mitochondrial and peroxisomal β-oxidation, were upregulated to levels comparable to those observed with the full agonist WY-14643 (Figure 1J) [41].

We also examined *CYP7A1*, which encodes cholesterol 7α-hydroxylase, the rate-limiting enzyme in bile acid biosynthesis. *CYP7A1* mRNA levels decreased dose-dependently following RT206 treatment, consistent with reported repression by PPARα signaling and FXR-mediated regulation of bile acid synthesis [12,42,43].

These data demonstrate that RT206 behaves as a balanced partial agonist in GAL4-PPAR transactivation assays and activates canonical PPAR target genes at endogenous loci in HepG2 cells. These findings confirm PPAR engagement and prompted investigation of whether RT206 might influence FXR-dependent transcription.

### 3.3. RT206 Binds FXR as Assessed by Grating-Coupled Interferometry (GCI) but Does Not Transactivate FXR

We investigated the potential binding of RT206 to FXR using GCI, a label-free technique based on principles analogous to surface plasmon resonance. For the GCI binding experiments, recombinant human FXR-LBD, custom-produced and purified in a bacterial expression system by Biogem S.c.ar.l., was immobilized on a polycarboxylate sensor chip by amine coupling, as described in Section 2. The analytical workflow followed established label-free interaction analysis procedures [44,45]. The chemical structures of RT206 and of the FXR orthosteric agonists GW4064 and OCA used throughout this study are shown in Figure 1K.

Kinetic analyses demonstrated a concentration-dependent binding of RT206 to FXR, with a calculated equilibrium dissociation constant (K_d_) in the millimolar range (Figure S2). Because this value reflects interaction with the unliganded LBD, it does not necessarily predict engagement of ligand-bound or conformationally stabilized receptor states. These data, therefore, indicate weak binding to apo-FXR and leave open the possibility that RT206 preferentially recognizes a structurally primed conformation.

**Figure 2:**
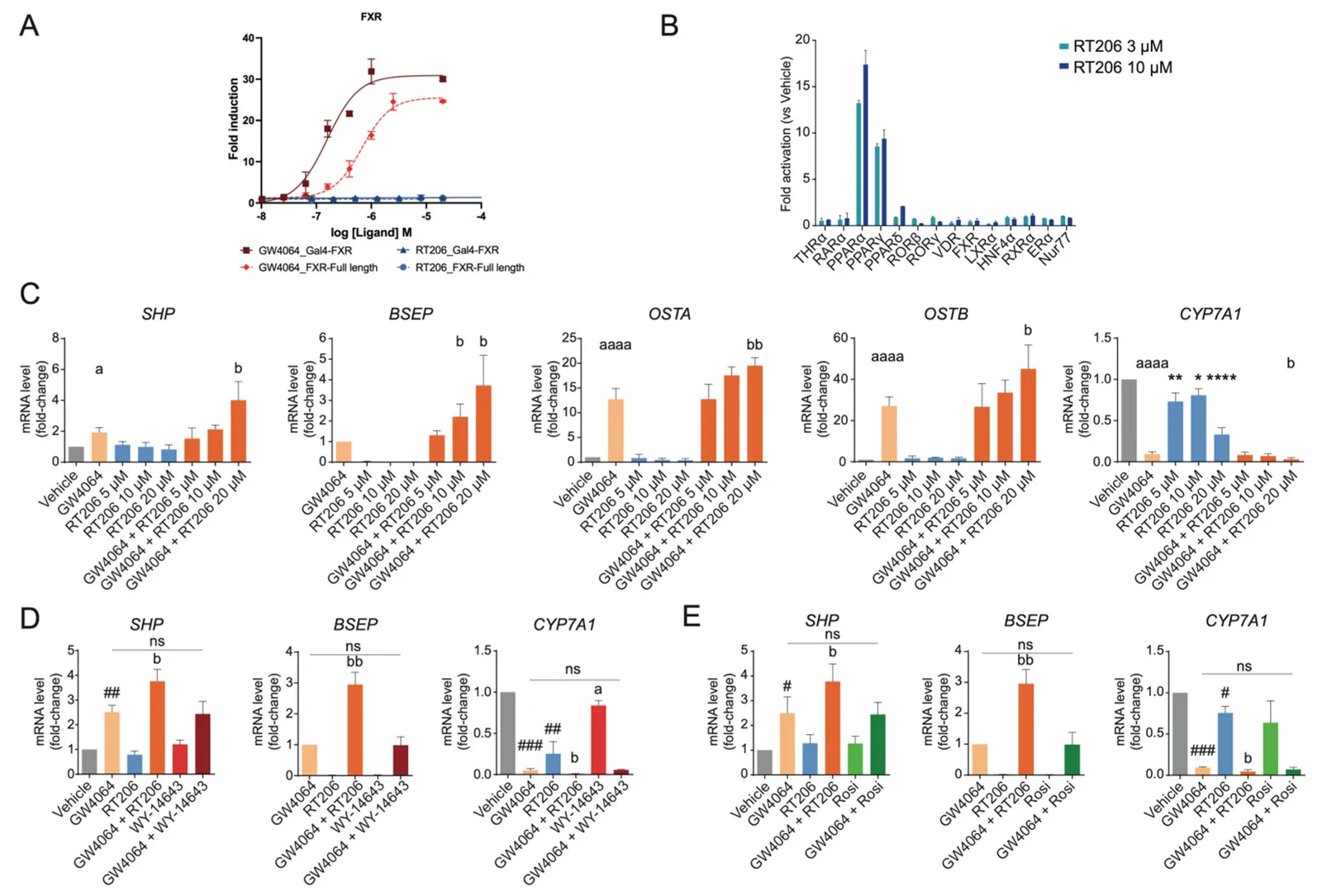
RT206 lacks intrinsic FXR agonist activity and potentiates FXR-dependent transcription in the presence of an orthosteric agonist in hepatoma cells. (**A**) RT206 does not activate FXR in transactivation assays using full-length FXR or a GAL4-FXR chimeric construct in HepG2 cells. GW4064 was used as positive control. (**B**) Selectivity profiling of RT206 (3 and 10 µM) across a panel of nuclear receptors in GAL4-based transactivation assays in HEK293T cells. Activation was detected only for PPARα and PPARγ. (**C**) Expression of *SHP* (n = 4), *BSEP* (n = 4), *OSTA* (n = 5), *OSTB* (n = 5), and *CYP7A1* (n = 4) genes in HepG2 cells treated for 24 h with the vehicle (DMSO) or GW4064 (1 µM), the indicated doses of RT206, alone or in combination. (**D**) Assessment of *SHP* (n = 3), *BSEP* (n = 3) and *CYP7A1* (n = 3) mRNAs in HepG2 cells treated for 24 h with the vehicle (DMSO) or GW4064 (1µM), RT206 (20 µM), WY-14643 (10 µM), alone or in combination. (**E**) Analysis of *SHP* (n = 4), *BSEP* (n = 3) and *CYP7A1* (n = 3) expression in HepG2 cells treated for 24 h with the vehicle (DMSO) or GW4064 (1 µM), RT206 (20 µM), or Rosiglitazone (Rosi, 1 µM), alone or in combination. All histograms report normalization of mRNA levels to 18S in RT-qPCR analysis. Only for *BSEP* expression, fold-change is referred to GW4064 treatment, as the basal level is very low or undetermined. Statistical significance was considered when * *p* < 0.05, ** *p* < 0.01, or **** *p* < 0.0001 (ANOVA with Dunnett’s post-test vs. vehicle); a *p* < 0.05, aaaa *p* < 0.0001 (*t*-test vs. vehicle); b *p* < 0.05 or bb *p* < 0.01 (*t*-test with Bonferroni correction vs. GW4064), # *p* < 0.05, ## *p* < 0.01, or ### *p* < 0.001 (*t*-test with Bonferroni correction vs. vehicle).

To address this question and to determine whether RT206 acts as a direct FXR agonist, we performed GAL4-FXR transactivation assays on transiently transfected HepG2 cells using increasing concentrations of the full agonist GW4064 or RT206. RT206 did not induce measurable FXR-dependent luciferase activation, whereas the positive control robustly activated the reporter (Figure 2A). To further rule out activation of unrelated nuclear receptors, RT206 (3 and 10 μM) was tested across a panel of GAL4-based chimeric nuclear receptor reporters transfected in HEK293T cells. Activation was restricted to PPARα and PPARγ, with no detectable activation of FXR or other receptors examined (Figure 2B), confirming the results obtained in HepG2 cells.

These findings demonstrate that RT206 does not function as an orthosteric FXR agonist in classical transactivation assays and suggest that its interaction with FXR may occur through a mechanism distinct from canonical ligand activation.

### 3.4. RT206 Potentiates FXR-Dependent Transcription in the Presence of Orthosteric Agonists

We thus evaluated whether RT206 modulates FXR activity in the presence of an orthosteric agonist. HepG2 cells were treated for 24 h with GW4064 (1 μM), RT206 (5–20 μM), or their combination, and FXR target genes were quantified with respect to vehicle-treated cells by RT-qPCR. *SHP*, a canonical FXR target gene mediating transcriptional repression of bile acid synthesis [12,46], was induced approximately two-fold by GW4064, whereas RT206 alone had no effect. Co-treatment significantly enhanced *SHP* expression compared to GW4064 alone, particularly at 10 and 20 μM RT206 (Figure 2C). *BSEP*, a direct FXR-dependent canalicular bile acid transporter [38,46], is expressed at low levels in HepG2 cells and was strongly induced by GW4064. RT206 alone was inactive; however, the combination elicited a marked additional increase, detectable at 10 and 20 μM RT206. Similarly, *OSTA* (Solute Carrier Family 51 subunit α) and *OSTB* (Solute Carrier Family 51 subunit β), encoding a heterodimeric transporter involved in basolateral bile acid secretion [46], were more strongly induced by the GW4064/RT206 combination than by GW4064 alone, while RT206 alone had no effect. *CYP7A1* expression was strongly reduced by GW4064 and modestly decreased by RT206 alone. Combined treatment produced a repression greater than that observed with either compound alone. Because *CYP7A1* is co-regulated by FXR- and PPARα-dependent pathways [12,42,43], the enhanced repression observed during co-treatment is compatible with concurrent modulation of FXR- and PPARα-dependent pathways. These data indicate that RT206 does not activate FXR in isolation but enhances FXR-dependent transcription in the presence of an orthosteric agonist, consistent with a positive allosteric modulator (PAM) behavior [47]. Importantly, the absence of FXR target induction by RT206 alone argues against intrinsic agonism and supports a context-dependent potentiation mechanism.

To assess whether the observed enhancement could result from indirect PPAR-mediated upregulation of FXR expression, HepG2 cells were treated with GW4064 alone or in combination with selective PPARα (WY-14643, 10 μM) or PPARγ (Rosiglitazone, 1 μM) agonists [48].

*SHP* and *BSEP* mRNA levels were induced by GW4064 but were not further enhanced by WY-14643 or Rosiglitazone (Figure 2D,E). *CYP7A1* was strongly downregulated by GW4064 and more modestly by RT206 or WY-14643 (Figure 2D), but not by Rosiglitazone (Figure 2E), consistent with contributions from FXR- and PPARα-dependent pathways under these conditions. The inability of selective PPAR agonists to reproduce the potentiation observed with RT206 argues against an indirect transcriptional cross-regulation mechanism and supports a receptor-level component of the observed modulation.

Furthermore, we determined that RT206-mediated potentiation was not restricted to GW4064 by treating HepG2 cells with OCA (10 μM) alone or in combination with RT206 (5–20 μM) (Figure S3A).

*SHP*, *BSEP*, *OSTA* and *OSTB* mRNA levels were induced by OCA and further enhanced by co-treatment with RT206. *CYP7A1* repression by OCA was also strengthened in combination. The preservation of cooperative enhancement with both a synthetic non-steroidal agonist and a steroidal bile acid-derived agonist indicates that RT206-mediated potentiation is not limited to a specific orthosteric scaffold.

Finally, to rule out that the variations in gene expression were due to non-specific transcriptional alterations secondary to cellular stress, we conducted cell viability assays on cells exposed to increasing concentrations of RT206 in combination with GW4064, OCA or the selective PPARα or PPARγ agonist, WY-14643 or Rosiglitazone, respectively. A modest cytotoxic effect was detected only at high concentrations (Figure S3B).

### 3.5. RT206 Binds to FXR and Displays Ligand-Dependent Cooperative Binding as Assessed by GCI

To test whether RT206 binds FXR in a non-competitive manner, we applied a protocol previously used to distinguish orthosteric competition from cooperative binding events in ligand-regulated transcription factors [49]. In this approach, binding signals obtained from individual ligands are compared to those obtained from their mixture. A combined signal lower than the arithmetic sum indicates competition, whereas an equal or higher signal indicates binding to distinct sites.

We evaluated RT206 binding to FXR in the presence of increasing concentrations of GW4064 or OCA. Orthosteric ligand concentrations were informed by the independently determined $K_{d}$ values of 51 nM for GW4064 and 4 µM for OCA and were selected to provide measurable sensor responses under the mixture conditions used in the assay (Figure S4). The mixture experiments were designed to assess whether orthosteric ligand occupancy affected the interaction of RT206 with FXR.

The mixture responses showed no evidence of competitive displacement. Instead, above-additive binding signals were observed under all three conditions examined, although their magnitude varied with the orthosteric ligand and concentration. In the presence of 100 µM RT206, the mixture signal exceeded the arithmetic sum of the individual responses by 121% with 5 µM GW4064 and by 24% with 10 µM GW4064. The combination of 20 µM OCA and 100 µM RT206 produced an 18% above-additive increase (Figure S5). These findings are consistent with simultaneous, non-competitive binding and support positive cooperativity between RT206 and orthosteric FXR ligands [50]. These mixture experiments were performed in a cell-free format on purified FXR-LBD and were intended to provide mechanistic information on ligand binding rather than to reproduce cellular exposure conditions.

To further investigate ligand-dependent effects, kinetic binding analyses of RT206 were performed in the presence or absence of fixed concentrations of orthosteric ligands. Addition of 1 µM GW4064 reduced the apparent RT206 dissociation constant from approximately 2.7 mM to 794 µM. Similarly, in the presence of 10 or 20 µM OCA, the K_d_ decreased to 1.9 mM and 831 µM, respectively (Figure S6). The decrease in apparent K_d_ in the presence of orthosteric ligands indicates agonist-dependent stabilization of RT206 binding, consistent with positive allosteric modulation [50]. The apparent Kd values determined by GCI refer to the isolated, immobilized FXR LBD under cell-free conditions and are not directly comparable to the concentrations used in cell-based functional assays.

**Figure 3:**
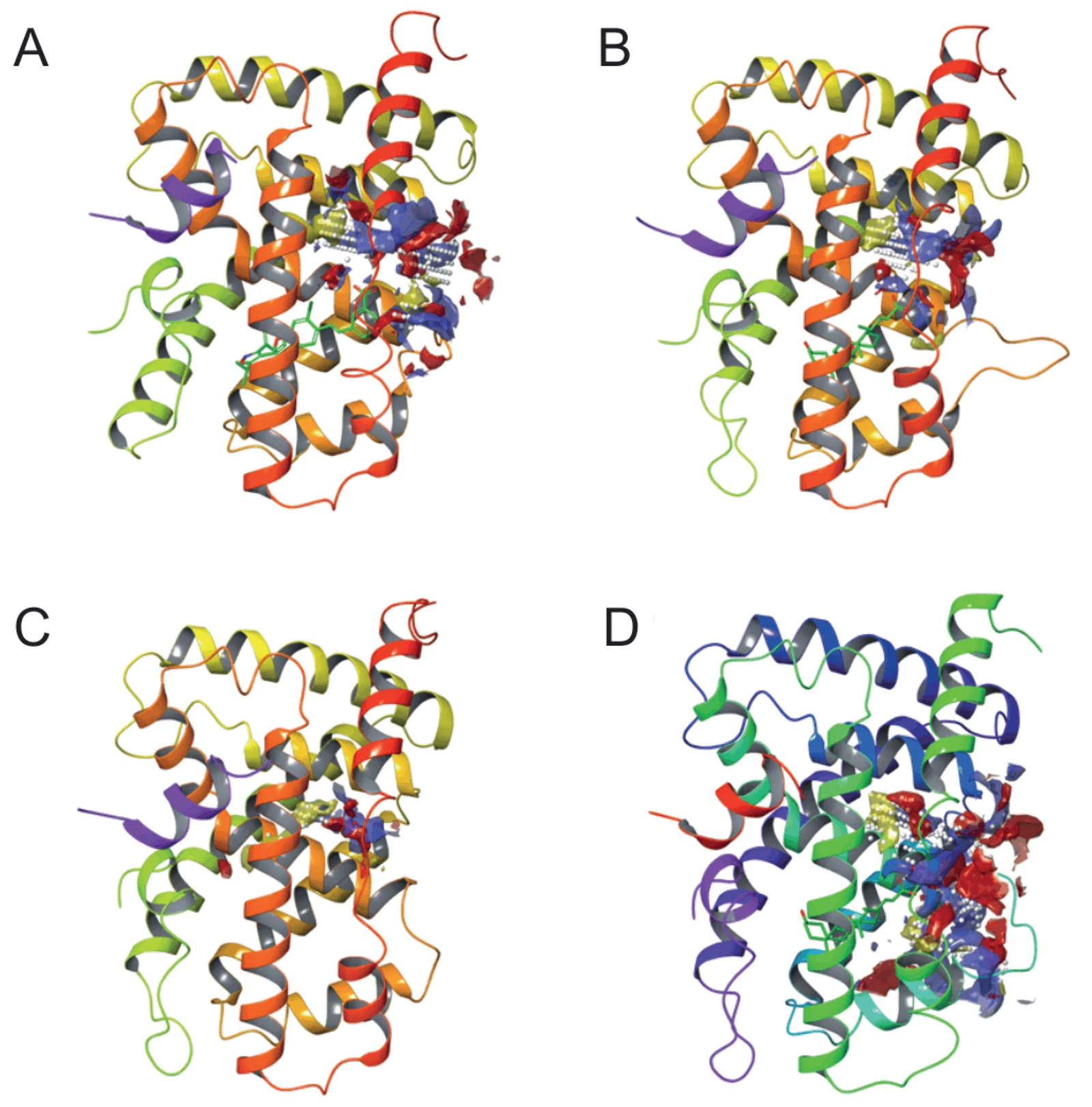
SiteMap analyses of the FXR-LBD across different receptor states. SiteMap-identified binding sites are shown as contour maps for FXR in complex with (**A**) the synthetic agonist GW4064 (PDB 3DCT), (**B**) the endogenous bile acid chenodeoxycholic acid (CDCA, PDB 6HL1), (**C**) the apo receptor (PDB 6HL0), and (**D**) OCA (PDB 1OSV, rat FXR). Corresponding SiteScore, Dscore and volume values are reported in Table S3. Differences in coloring reflect species-specific structural representation.

We were unable to perform reciprocal kinetic analyses of GW4064 or OCA in the presence of fixed RT206 concentrations, because the RT206 levels required to achieve measurable occupancy exceeded the practical assay window and solubility limits. Together, these results indicate that RT206 engages FXR through a non-competitive and ligand-enhanced binding mechanism consistent with positive allosteric modulation. The structural basis of this non-competitive interaction is examined in the following section (Figure 3A–D).

### 3.6. Molecular Docking and Dynamics Support a Model of RT206 Binding to the FXR S2 Pocket

To identify potential non-orthosteric binding sites, we analyzed representative FXR crystal structures in apo and agonist-bound conformations (see Section 2 for PDB entries), accounting for ligand-induced conformational variability [28,29,31]. Binding site identification was performed using SiteMap (Schrödinger Release 2025-4), which ranks pockets based on geometric and physicochemical descriptors summarized into SiteScore and Druggability Score (Dscore). We applied standard SiteMap thresholds to identify potentially druggable cavities and prioritized pockets recurrent across multiple receptor conformations. Comparative analysis revealed a recurrent cavity adjacent to the H1–H2 loop, distinct from the canonical orthosteric site S1, in the GW4064-bound (Figure 3A), CDCA-bound (Figure 3B), apo (Figure 3C) and OCA-bound rat (Figure 3D) receptor. This pocket displayed favorable druggability metrics and conformational variability (Table S3), consistent with structural plasticity. The identified region overlaps with residues previously implicated in the so-called S2 or “backdoor” site of FXR, proposed to modulate receptor activity. Hydrogen–deuterium exchange, modeling, and mutagenesis studies have highlighted the H1–H2 region as functionally relevant, including a threonine hotspot (T396 in α4 isoform numbering, corresponding to T386 in the structures used here) in S2-focused investigations [51,52,53,54]. These precedents supported explicit evaluation of RT206 within S2.

Docking was performed on FXR/GW4064/RT206 and FXR/OCA/RT206 ternary complexes to reproduce GCI experimental conditions. RT206 was placed in the SiteMap-identified S2 cavity in the presence of the orthosteric ligand, and top-ranked poses were subjected to MD simulations followed by clustering analysis. The full set of simulated systems is summarized in Table S4.

**Figure 4:**
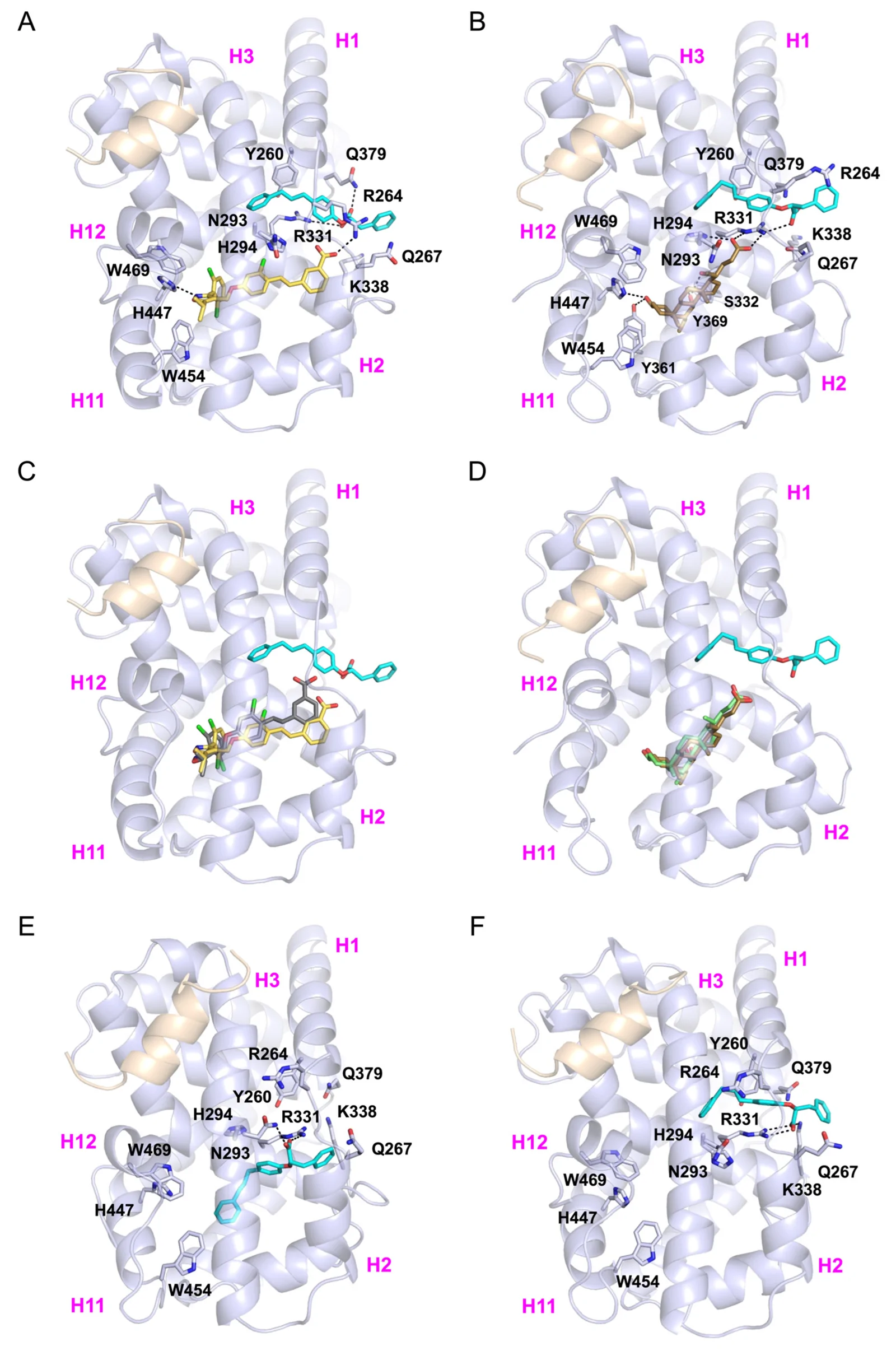
Predicted RT206 binding modes in FXR ternary assemblies with GW4064 or OCA and in binary complexes from 1 μs MD simulations. (**A**) FXR/GW4064/RT206 ternary complex. The orthosteric agonist GW4064 is shown as yellow sticks and RT206 as cyan sticks. (**B**) FXR/OCA/RT206 ternary complex. The orthosteric agonist OCA is shown as tan sticks and RT206 as cyan sticks. (**C**) Structural overlay of the GW4064 crystallographic pose (gray sticks; PDB 3DCT) with the ternary FXR/GW4064/RT206 complex. (**D**) Structural overlay of the OCA crystallographic pose (green sticks; PDB 1OSV) with the ternary FXR/OCA/RT206 complex. (**E**) Predicted RT206 binding mode in the orthosteric S1 site of the binary FXR/RT206 complex. (**F**) Predicted RT206 binding mode in the S2 backdoor pocket of the binary FXR/RT206 complex. In all panels, the FXR-LBD is rendered as a light blue cartoon, the coactivator peptide is shown in wheat, and key interacting residues are shown as white sticks with corresponding labels. Relevant helices are indicated in magenta. Hydrogen bonds are depicted as dashed black lines. All MD-derived structures shown are representative frames of the most populated cluster (see Section 2 for clustering details and Table S4 for the simulated systems).

Figure 4 shows the representative binding modes extracted from the most highly populated cluster for each ternary complex. In both ternary systems, RT206 remained stably accommodated within the S2 pocket throughout the full MD trajectories, forming a persistent polar interaction network. The dominant anchoring interaction involved R331, which consistently engaged RT206 and acted as the principal electrostatic anchor in the simulated complexes. In the FXR/GW4064/RT206 complex (Figure 4A), additional hydrogen bonding with Q379 and R264 reinforced stabilization. GW4064 preserved its canonical binding mode, including interactions with H447, W469 and W454, and maintained contacts consistent with proper helix 12 positioning (Figure 4C).

In the FXR/OCA/RT206 complex (Figure 4B), RT206 again adopted a comparable S2 binding orientation and maintained interaction with R331. Concurrently, OCA occupied the orthosteric S1 site, with its carboxylate forming stable polar contacts with R331 and H294, the 7α-OH hydrogen-bonding to S332 and Y369, and the 3α-OH interacting with Y361 and H447, collectively stabilizing the canonical agonist interaction network (Figure 4D). The coexistence of RT206 in S2 with orthosteric ligands in S1 offers a possible structural rationale for the experimentally observed non-competitive binding behavior [49,52].

To rationalize the agonist-enhanced RT206 affinity observed experimentally, comparative docking and MM-GBSA calculations [55] were performed for RT206 in S1 and S2 as a standalone ligand.

The S1 pose (Figure 4E) showed strong interactions with R331 and N293 but failed to engage W469, H447, and W454 effectively. Although MM-GBSA yielded a highly favorable binding energy (−77.88 kcal/mol), this configuration is inconsistent with the absence of competitive binding and the lack of agonist activity in cellular assays. Thus, the S1 placement likely represents a computationally favorable but functionally implausible configuration.

In contrast, RT206 binding within S2 resulted in a more moderate binding energy (−57.69 kcal/mol), consistent with the weak affinity measured for apo-FXR (Figure 4F). Formation of the ternary FXR/OCA/RT206 complex produced a modest energetic improvement (−60.26 kcal/mol), paralleling the experimentally observed ligand-dependent stabilization.

Together, these structural and energetic analyses support a model in which orthosteric ligand binding stabilizes an FXR conformation compatible with productive engagement of RT206 within the S2 pocket. The combined gene expression, GCI, and computational data converge on a positive allosteric mechanism whereby RT206 coexists with orthosteric ligands and enhances receptor output without competing for the S1 site.

### 3.7. RT206 and Guggulsterone (GG) Differentially Affect FXR Gene Expression

RT206 appears to interact with the S2 region, previously described as a “backdoor” pocket capable of modulating orthosteric ligand responses. Among reported S2-interacting ligands, GG has been described as an FXR antagonist [56] and as a modulator of receptor conformation through the S2 pocket [52,53,57]. We therefore used GG as a benchmark S2 ligand to compare binding cooperativity and transcriptional consequences with RT206.

**Figure 5:**
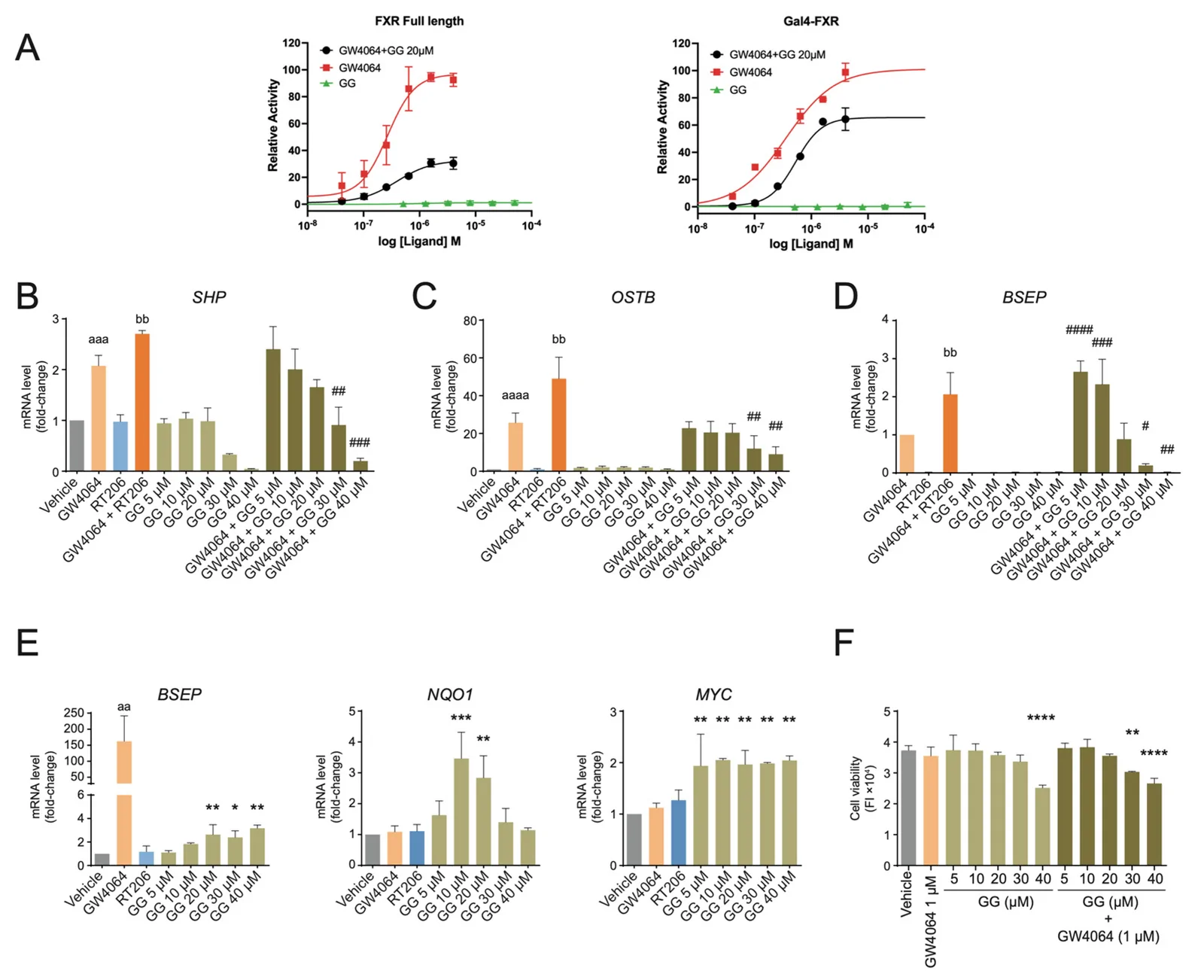
Guggulsterone (GG) acts as an FXR antagonist in transactivation and gene expression assays in hepatoma cells. (**A**) Dose–response curves of GG on FXR full-length (left panel) and FXR-GAL4 chimera (right panel) compared to the full agonist GW4064. (**B**) *SHP* (n = 3), (**C**) *OSTB* (n = 4), and (**D**) *BSEP* (n = 4) mRNA levels measured by RT-qPCR in HepG2 cells treated for 24 h with vehicle (DMSO) or GW4064 (1 µM), the indicated doses of GG, alone or in combination. Cells treated with GW4064 (1 µM) and RT206 (20 µM), alone or in combination, served as controls. Only for *BSEP* expression, fold-change is referred to GW4064 treatment, as the basal level is very low or undetermined. (**E**) *BSEP* (n = 4), *NQO1* (n = 3) and *MYC* (n = 3) mRNA levels in HepG2 cells treated for 24 h with vehicle (DMSO) or GW4064 (1 µM), RT206 (20 µM) or increasing doses of GG. (**F**) Viability (n = 3) of HepG2 cells treated for 24 h with the indicated concentrations of GG alone or in combination with GW4064 (1 µM). All histograms report normalization of mRNA levels to 18S in RT-qPCR analysis. Statistical significance was considered when aa *p* < 0.01, aaa *p* < 0.001, or aaaa *p* < 0.0001 (*t*-test vs. vehicle); bb *p* < 0.01 (*t*-test vs. GW4064). # *p* < 0.05, ## *p* < 0.01, ### *p* < 0.001, or #### *p* < 0.0001 (ANOVA with Dunnett’s post-test vs. GW4064); * *p* < 0.05, ** *p* < 0.01, *** *p* < 0.001, or **** *p* < 0.0001 (ANOVA with Dunnett’s post-test vs. vehicle).

Firstly, GG activity was validated in co-transfection assays. As expected, GG behaved as an FXR antagonist, showing dose-dependent inhibition of GW4064-mediated FXR activation in both full-length and chimeric receptor systems, with IC_50_ values of 6.1 ± 0.5 μM and 22.1 ± 0.7 μM, respectively (Figure 5A).

GCI experiments showed that GG binds FXR with a K_d_ of 549 µM (Figure S7A). To assess competition versus cooperativity, GG was tested alone or in combination with GW4064 at different concentration ratios. At 10 µM GW4064 and 100 µM GG, the combined signal exceeded the arithmetic sum of individual signals by 27% (Figure S7B), consistent with non-competitive binding and ternary complex formation [49]. Importantly, above-additive binding does not equate to positive allosteric modulation at the transcriptional level but indicates simultaneous site occupancy under these experimental conditions.

Given the similar GCI profile between GG and RT206, we evaluated whether FXR-dependent gene expression was likewise influenced in HepG2 cells treated with 1 µM GW4064 and increasing concentrations of GG (5–40 µM), alone or in combination.

*SHP* mRNA was induced by GW4064 but not by GG alone. At 5–10 µM GG, co-treatment produced *SHP* induction comparable to GW4064 alone, whereas higher GG concentrations dose-dependently attenuated GW4064-mediated stimulation (Figure 5B).

*OSTB* expression reproduced the results of *SHP*: GW4064 induced a significant elevation, while GG had no effect alone and, in combination, dose-dependently reduced the GW4064-elicited stimulation (Figure 5C). *BSEP* expression is low in HepG2 cells and was robustly increased by GW4064 but only slightly by 20 µM GG and above (Figure 5D,E). The combination of GW4064 with 5 and 10 µM doses of GG elicited a higher stimulation, while, in contrast, 20, 30 and 40 µM doses produced a dose-dependent reduction to almost undetectable levels (Figure 5D). As controls, cells were also treated with GW4064 (1 µM), RT206 (20 µM), or their combination, and the results confirmed those reported above (Figure 5B–D).

These results show that RT206 reproducibly potentiates orthosteric agonist-dependent regulation of the FXR target genes examined, whereas GG displays a dose-dependent biphasic profile, with apparent enhancement at low concentrations and antagonism at higher concentrations [52]. This divergent behavior suggests that interaction with the proposed non-orthosteric region does not by itself determine functional outcome and that transcriptional responses are ligand- and concentration-dependent.

To clarify the basis of this biphasic behavior, we investigated whether GG regulates *BSEP* through FXR-independent mechanisms. *BSEP* transcription has been reported to be regulated by GG via the transcription factor AP-1, which binds a functional site within the human promoter in addition to the FXR-responsive IR1 element [58]. This dual promoter architecture provides a mechanistic basis for transcription factor cooperation and context-dependent modulation independently of classical FXR transactivation [40,58,59].

HepG2 cells were therefore treated with increasing GG concentrations (5–40 µM) for 24 h, and AP-1 target genes were evaluated. *BSEP* expression was slightly induced while *NQO1* mRNA, encoding NAD(P)H:quinone oxidoreductase, was robustly induced at 10 µM GG and declined at higher doses (Figure 5E) [58,60,61]. Similarly, *MYC*, another AP-1-regulated gene, was consistently induced by GG from 5 µM onward. Neither of the latter genes was stimulated by GW4064 or RT206 (Figure 5E), supporting distinct mechanistic modes of action for the two S2-interacting ligands. These data indicate that low-dose enhancement of *BSEP* by GG likely reflects superimposed AP-1-mediated transcription, whereas higher concentrations favor antagonistic FXR modulation. In contrast, RT206 consistently potentiates FXR-dependent transcription without activating AP-1-responsive genes.

Finally, cell viability was assessed after exposure to increasing concentrations of GG alone or in combination with GW4064. Viability was largely preserved up to 20 µM GG, whereas reductions were observed at higher concentrations, particularly in the presence of GW4064 (Figure 5F). These findings indicate that the transcriptional effects observed at the lower GG concentrations are unlikely to result from nonspecific cytotoxicity.

Overall, RT206 behaves as a consistent positive allosteric modulator across the FXR targets examined, whereas GG displays gene- and dose-dependent effects consistent with the proposed S2-binding model and parallel AP-1-mediated transcriptional regulation.

### 3.8. Comparative In Silico Analysis Provides Insights into the Modulatory Effects of GG on FXR

Prompted by the GCI results and the established role of the S2 pocket as a GG binding site, we extended our *in silico* analysis to directly compare the binding signatures of GG and RT206, aiming to rationalize their distinct transcriptional outcomes. Because of the relatively rigid steroidal scaffold of GG, minor adaptations to the computational workflow were implemented to ensure adequate exploration of the S2 pocket (see Section 2 and Table S4). All simulations were performed using the same orthosteric ligands and docking/MD workflow described above to enable direct comparison of ternary complexes. All trajectories reached stable RMSD plateaus within the first 300 ns and displayed reproducible behavior across independent replicas (Figure S1), consistent with well-equilibrated production sampling.

Throughout the simulations, GG remained stably accommodated within the S2 pocket, predominantly stabilized by hydrophobic contacts. However, unlike RT206, GG induced a marked conformational displacement of the orthosteric agonists relative to their crystallographic poses, consistent with previous reports of GG-induced conformational perturbations of the FXR ligand-binding domain [51].

**Figure 6:**
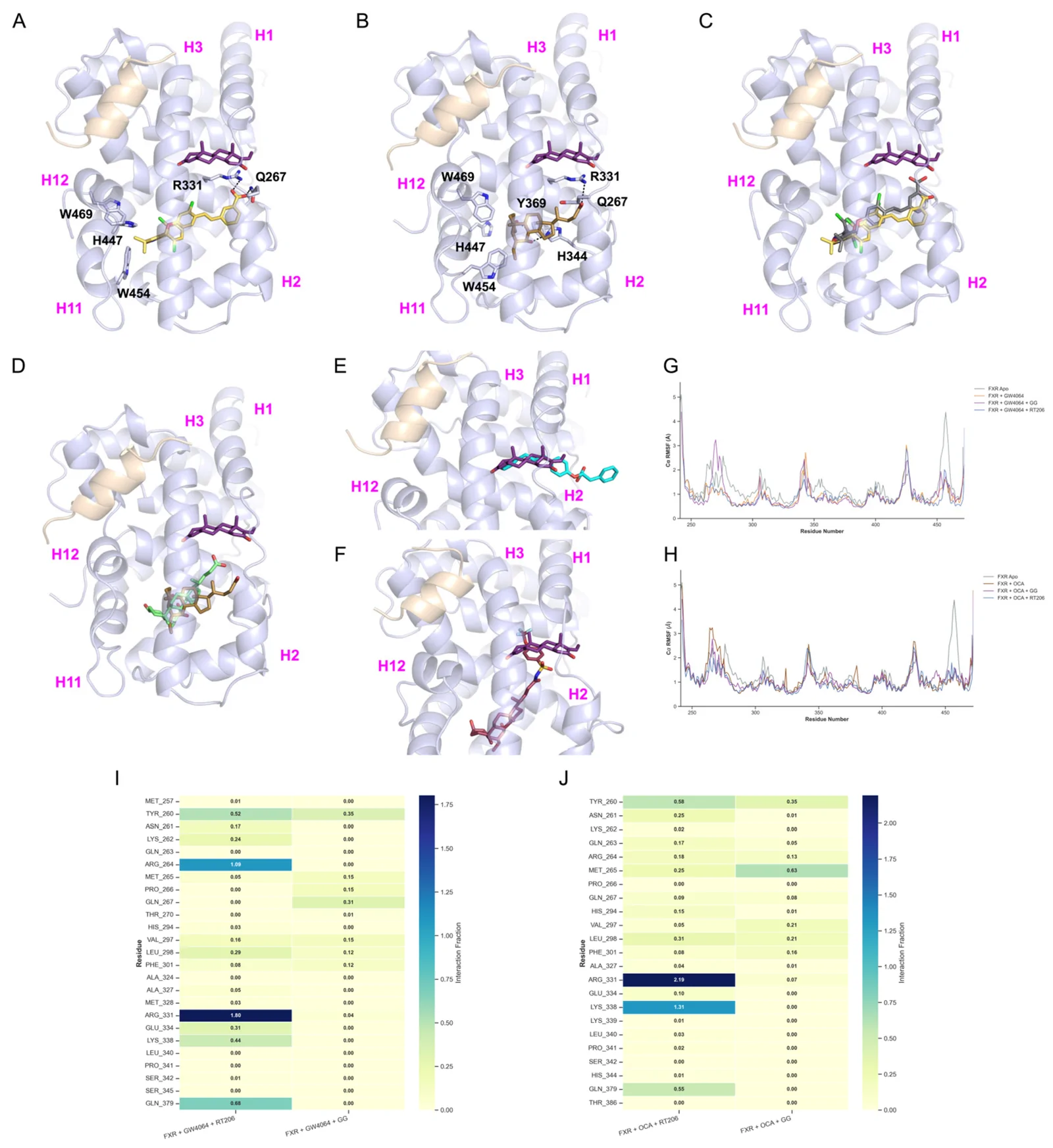
Predicted GG binding modes in FXR ternary complexes with GW4064 or OCA from 1 μs MD simulations and corresponding S2-pocket interaction maps. (**A**) FXR/GW4064/GG ternary complex. The orthosteric agonist GW4064 is shown as yellow sticks and GG as purple sticks. (**B**) FXR/OCA/GG ternary complex. The orthosteric agonist OCA is shown as tan sticks and GG as purple sticks. (**C**) Structural overlay of the GW4064 crystallographic pose (gray sticks; PDB 3DCT) with the ternary FXR/GW4064/GG complex. (**D**) Structural overlay of the OCA crystallographic pose (green sticks; PDB 1OSV) with the ternary FXR/OCA/GG complex. (**E**) Overlay of GG and RT206 (cyan sticks) bound to the FXR LBD. (**F**) Overlay of GG and compound **2** (firebrick sticks, PDB 9H66). Root mean square fluctuations (RMSFs) of FXR-LBD Cα atoms throughout the MD trajectories of the systems referred to in (**G**)—FXR/GW4064/RT206, FXR/GW4064/GG, FXR/GW4064 and the apo-receptor—or in (**H**)—FXR/OCA/RT206, FXR/OCA/GG, FXR/OCA and the apo-receptor. Interaction fraction heatmaps quantifying the frequency and persistence of contacts within the S2 pocket for RT206 and GG in ternary systems with (**I**) GW4064 or (**J**) OCA. The interaction fraction represents the normalized sum of hydrogen bonds, ionic interactions, hydrophobic contacts, and water-mediated bridges over the 1 μs simulation; values >1.0 denote residues engaged in multiple or highly stable simultaneous interactions. MD-derived structures in panels (**A**–**F**) are representative frames of the most populated cluster (see Section 2 for clustering details and Table S4 for the simulated systems).

In the FXR/GW4064/GG ternary complex, S2 occupancy by GG caused repositioning of the GW4064 isoxazole moiety. Although the interaction with R331 was retained, a new hydrogen bond formed between the GW4064 carboxylate and Q267 (Figure 6A,C), indicating alteration of the canonical interaction network. A similar effect was observed in the FXR/OCA/GG system (Figure 6B,D), where OCA lost part of its characteristic orthosteric interaction pattern and adopted an alternative orientation. Its carboxylate remained engaged with R331 but established a novel contact with H344. These orthosteric rearrangements suggest that GG binding at S2 can reshape the S1 binding environment, providing a structural explanation for its context-dependent antagonistic profile [54]. This orthosteric drift offers a structural correlate with the reduction of *SHP* and *BSEP* induction observed at higher GG concentrations.

Structural comparison of the RT206- and GG-bound complexes revealed partial spatial overlap between the steroidal core of GG and the hydrophobic tail of RT206 within the S2 pocket (Figure 6E). However, unlike RT206, GG did not establish persistent and directional polar interactions with key S2 residues. In particular, GG lacked stable electrostatic anchoring comparable to the R331-centered network observed for RT206, which may underlie its weaker stabilization of the active receptor conformation. Within the simulated complexes, these findings suggest that predicted S2 occupancy alone may not determine functional outcome and that interaction persistence and directionality may contribute to the distinct effects of the two ligands. Interestingly, both ligands occupy a region overlapping with the trifluoromethoxy group of compound **2** (Figure 6F), a steroidal modulator reported to explore the S2 backdoor via an extended side chain [62]. This overlap supports the structural versatility of S2 in accommodating chemically diverse scaffolds capable of modulating orthosteric efficacy.

To further dissect dynamic differences, we analyzed the root mean square fluctuation (RMSF) of Cα atoms across MD trajectories, comparing ternary complexes (FXR/GW4064/RT206, FXR/OCA/RT206, FXR/GW4064/GG, and FXR/OCA/GG) with their corresponding binary agonist complexes and apo-FXR. Apo-FXR (gray line) exhibited higher global flexibility, particularly in the C-terminal region encompassing helix 12 (residues ~450–460). As expected, binding of GW4064 or OCA reduced overall fluctuations (Figure 6G,H), reflecting agonist-induced stabilization of the active conformation [31].

Addition of RT206 produced a further decrease in RMSF values across the protein sequence (blue lines), indicating enhanced stabilization of the ligand-binding domain. In both GW4064 and OCA ternary complexes, RT206 reduced mobility of the H1–H2 loop and helix 12, suggesting reinforcement of the agonist-stabilized active conformation.

In contrast, GG induced a more heterogeneous fluctuation profile (purple line, Figure 6G,H). In the GW4064 series, GG increased local flexibility around residue ~270 within the H2 loop.

This localized destabilization, together with preserved but rearranged orthosteric interactions, supports a model in which GG perturbs S1 geometry rather than reinforcing the agonist-bound state.

With respect to the C-terminal region and helix 12 (residues 450–470), the transition from the apo state to RT206-containing ternary complexes was characterized by a marked collapse in RMSF, yielding the lowest residual fluctuations in H12 and consistent with reinforced stabilization of the active conformation.

Finally, interaction fraction analysis (Figure 6I,J) shows clear differences between the two ligands. RT206 formed a dense and persistent interaction network, characterized by high interaction fractions with R331 (>1.8) and consistent contacts with Q379 and R264 or K338. In contrast, GG exhibited predominantly transient hydrophobic interactions, including contacts with Y260 and M265, and lacked robust electrostatic anchors. This reduced interaction persistence correlates with the weaker conformational stabilization observed for GG and aligns with its antagonistic transcriptional profile.

Taken together, the gene-expression and molecular modeling data suggest that engagement of a common non-orthosteric region is not sufficient to predict the direction of FXR modulation. RT206 consistently enhanced orthosteric agonist-dependent regulation of the FXR target genes examined, whereas GG produced gene- and dose-dependent effects, including attenuation of *SHP*, *OSTB*, and *BSEP* induction at higher concentrations and activation of AP-1-responsive genes. In the simulated ternary complexes, these divergent functional profiles were associated with distinct interaction patterns: RT206 formed a persistent polar and ionic network centered on R331 while preserving the canonical geometry of the orthosteric agonists, whereas GG relied predominantly on hydrophobic contacts and was accompanied by orthosteric ligand rearrangement and greater local flexibility. Although these findings do not establish a causal structure–function relationship, they provide a plausible molecular framework linking ligand-specific S2 interaction patterns to divergent transcriptional outcomes.

### 3.9. RT206 Retains PPAR Activity and Ligand-Dependent FXR Potentiation in a Sequential In Vitro Steatosis Model

Finally, we evaluated whether the ability of RT206 to activate PPARs and potentiate FXR-mediated transcriptional signaling, observed under basal conditions, is preserved in a model of fatty acid-overloaded cells. We employed a well-established system based on HepG2 cells exposed for 24 h to a mixture of sodium oleate (O) and sodium palmitate (P) in a 2:1 ratio, which recapitulates key hepatocellular features of lipid accumulation and lipotoxic stress. Fatty acids were delivered to cells using a 0.67% bovine serum albumin (BSA)-containing vehicle (see Section 2), commonly used for fatty acid solubilization [63,64,65].

**Figure 7:**
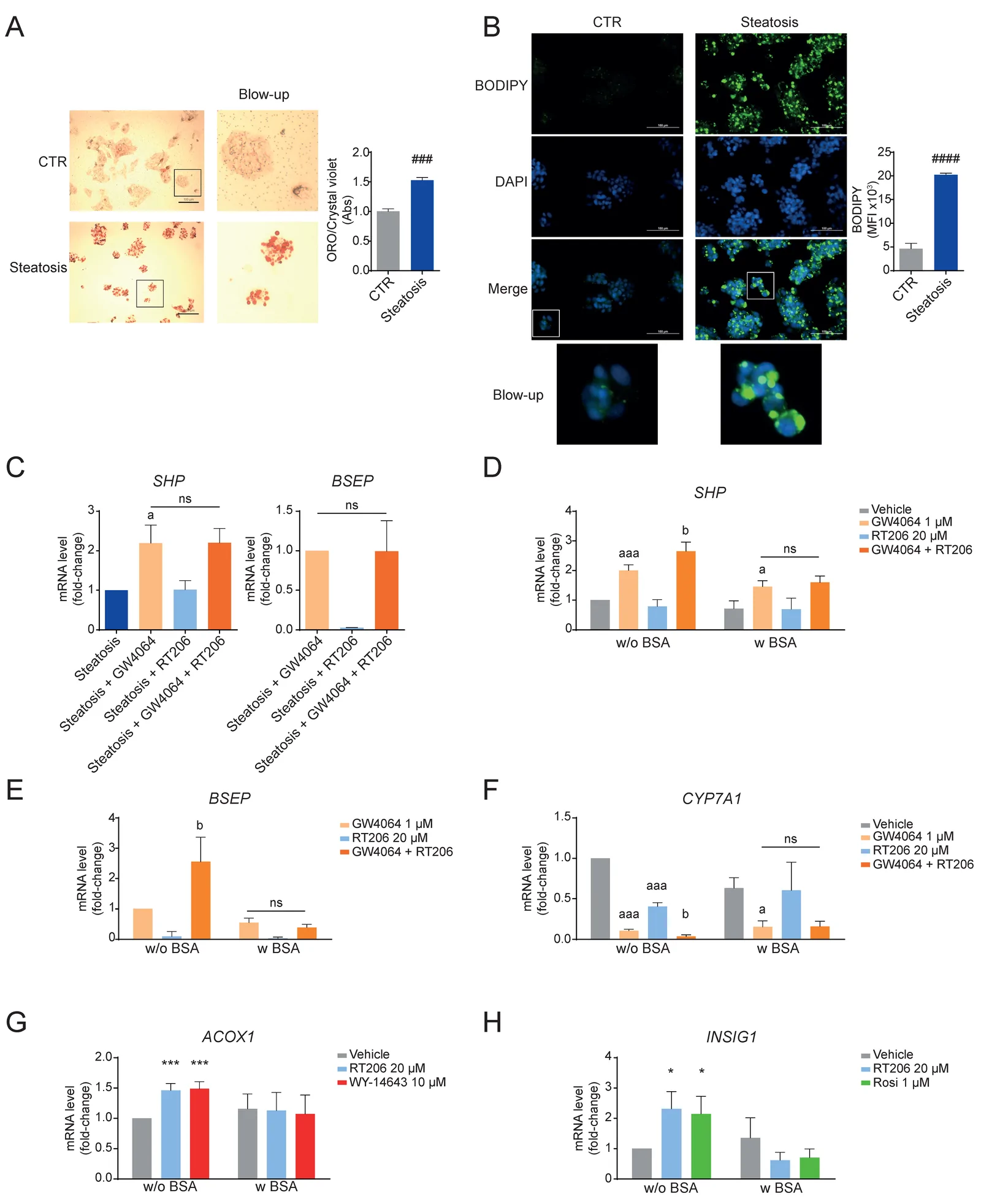
BSA attenuates RT206-dependent FXR and PPAR responses in a concurrent fatty acid-loading model. (**A**) Oil Red O (ORO) staining of HepG2 cells untreated (CTR) or treated with the fatty acid mixture (Steatosis) for 24 h (n = 3); the blow-ups on the side correspond to the indicated insets; the histogram reports the quantification of neutral lipid droplets as the ratio of ORO to crystal violet absorbance (20× objective, scale bar 100 µm). (**B**) BODIPY staining and fluorescence microscopy analysis of HepG2 cells treated as in (**A**) (n = 3). The panels show the BODIPY staining in green, nuclear staining with DAPI in blue and the merged image (20× objective, scale bar 100 µm, 1.5× magnification). The blow-ups at the bottom correspond to the indicated insets; the histogram reports the BODIPY signal captured by flow cytometry in the FITC channel and reported as MFI. (**C**) Assessment of *SHP* (n = 3) and *BSEP* (n = 3) expression in HepG2 cells treated with the fatty acid mixture (Steatosis) along with GW4064 (1 µM), RT206 (20 µM), alone or in combination, for 24 h. (**D**–**F**) *SHP* (n = 4) (**D**), *BSEP* (n = 3) (**E**), and *CYP7A1* (n = 3) (**F**) mRNA levels in HepG2 cells treated with GW4064 (1 µM), RT206 (20 µM), alone or in combination, for 24 h in the absence (w/o BSA) or presence (w BSA) of bovine serum albumin (BSA). Only for *BSEP* expression (**C**,**E**), fold-change is referred to GW4064 treatment, as the level in steatotic cells is very low or undetermined. (**G**) *ACOX1* expression (n = 4) in HepG2 cells treated with RT206 (20 µM) or the PPARα agonist WY-14643 (10 µM) for 24 h in the absence or presence of BSA. (**H**) *INSIG1* expression (n = 3) in HepG2 cells treated with RT206 (20 µM) or the PPARγ agonist Rosiglitazone (1 µM) for 24 h in the absence or presence of BSA. 18S RNA was used for mRNA normalization in RT-qPCR analysis. Statistical significance was considered when ### *p* < 0.001, or #### *p* < 0.0001 (*t*-test vs. CTR); a *p* < 0.05, aaa *p* < 0.001 (*t*-test with Bonferroni correction vs. Steatosis (Panel **C**) or vehicle (Panels **D**–**F**)); b *p* < 0.05 (*t*-test vs. GW4064 (Panels **D**–**F**)); * *p* < 0.05, or *** *p* < 0.001 (ANOVA with Dunnett’s post-test vs. vehicle (Panels **G**,**H**)).

Steatosis induction was verified by Oil Red O (ORO) staining, revealing abundant intracellular accumulation of neutral lipid droplets in treated cells, quantified by spectrophotometry and reported in the corresponding histogram (Figure 7A). BODIPY staining provided independent fluorescent confirmation of lipid accumulation, with increased granule abundance in treated versus control cells (Figure 7B). The concordance between ORO and BODIPY staining confirmed robust lipid loading prior to pharmacological intervention.

HepG2 cells were then treated for 24 h with the fatty acid mixture alone (steatotic cells) or together with GW4064 (1 µM), RT206 (20 µM), or their combination, given the established roles of FXR and PPARs in bile acid and lipid metabolism. Under these conditions, *SHP* and *BSEP* mRNAs were modestly induced by GW4064 but not by RT206. Notably, the combination GW4064/RT206 did not enhance gene expression beyond GW4064 alone (Figure 7C), indicating loss of the cooperative transcriptional effect observed under basal conditions.

We hypothesized that this discrepancy reflected reduced intracellular availability of RT206 due to BSA binding. To test this, cells were exposed for 24 h to 0.67% BSA alone, with or without GW4064 (1 µM), RT206 (20 µM), or their combination. Cells cultured without BSA served as controls, thereby isolating the effect of albumin from lipid loading.

In BSA-treated cells, *SHP* expression was not affected by RT206, only slightly induced by GW4064, and not further increased by the combination GW4064/RT206 (Figure 7D). *BSEP* mRNA, undetectable in vehicle-treated cells, was not affected by RT206, was minimally stimulated by GW4064 alone and was not further increased by the combination (Figure 7E). *CYP7A1* expression, which remained at basal levels with RT206, showed a moderate reduction with GW4064 and no additional effect with the GW4064/RT206 combination (Figure 7F). In cells exposed to regular medium without BSA, all these mRNAs were regulated as reported above.

Thus, BSA alone reproduces the loss of GW4064/RT206 cooperativity observed in steatotic conditions, consistent with a vehicle-dependent reduction in functional compound availability.

Because RT206 appeared particularly sensitive to the presence of BSA, we next examined whether PPAR activation was similarly attenuated by measuring *ACOX1* and *INSIG1*, representative PPARα- and PPARγ-regulated genes, respectively.

Cells were additionally treated with WY-14643 (10 µM) or Rosiglitazone (1 µM), reference agonists for PPARα and PPARγ, respectively. Neither *ACOX1* nor *INSIG1* was induced in the presence of BSA, whereas both genes were upregulated in BSA-free medium by RT206 and by the corresponding reference agonist (Figure 7G,H). These findings indicate that BSA attenuated both RT206-dependent PPAR activation and the responses to the reference PPAR agonists under the conditions tested.

These observations are consistent with the well-documented ability of serum albumin to bind hydrophobic small molecules and reduce their effective free concentration in cell culture systems [66]. Accordingly, the apparent loss of cooperativity in the standard steatosis protocol is best interpreted as a vehicle-dependent exposure limitation rather than a loss of intrinsic RT206 activity under metabolic stress.

To separate lipid loading from pharmacological exposure, HepG2 cells were first incubated with the BSA-containing fatty acid mixture for 24 h. The medium was then replaced with fatty acid- and BSA-free medium containing GW4064, RT206, or their combination for an additional 24 h. We first verified that intracellular lipid accumulation persisted after this washout period by comparing untreated cells, cells loaded with fatty acids for 24 h, and cells subjected to the sequential 24 h + 24 h protocol.

**Figure 8:**
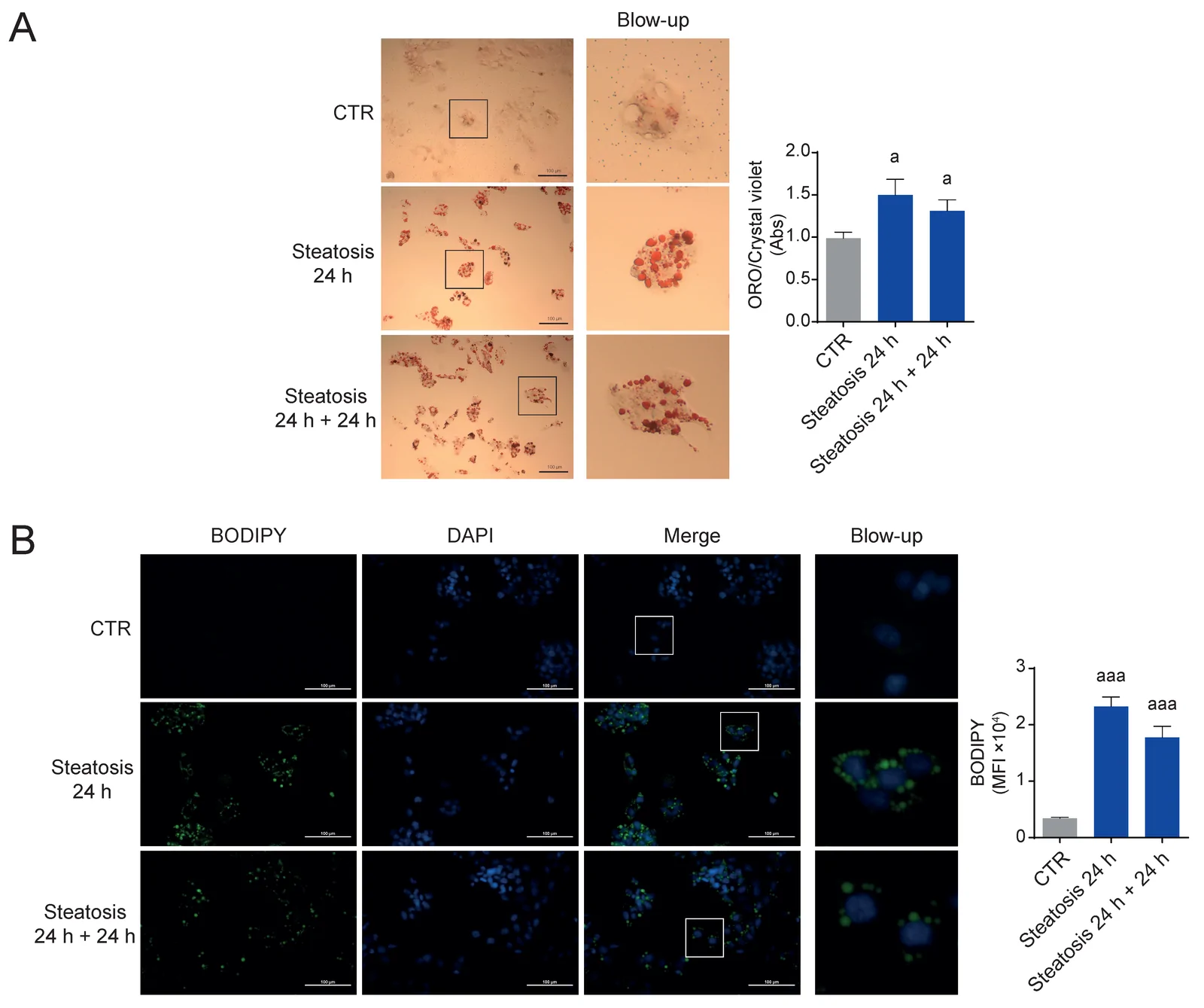
Characterization of neutral-lipid accumulation in the classical and sequential fatty acid-loading models. (**A**) Oil Red O staining of untreated HepG2 cells (CTR), cells exposed to the fatty acid mixture for 24 h (Steatosis 24 h), or cells exposed to the fatty acid mixture for 24 h followed by 24 h in fatty acid- and BSA-free medium (Steatosis 24 h + 24 h) (n = 3). The enlarged images correspond to the indicated insets. The histogram reports neutral-lipid accumulation as the ratio of Oil Red O to crystal violet absorbance. Images were acquired using a 20× objective; scale bar, 100 µm. (**B**) BODIPY fluorescence microscopy of cells treated as in A. BODIPY-stained neutral lipids are shown in green, nuclei stained with DAPI in blue, and merged images in the corresponding column. Enlarged images correspond to the indicated insets. The histogram reports BODIPY mean fluorescence intensity (MFI) measured by flow cytometry in the FITC channel (n = 3). Images were acquired using a 20× objective; scale bar, 100 µm; 1.5× magnification. Statistical significance was considered when a *p* < 0.05, or aaa *p* < 0.001 (*t*-test with Bonferroni correction vs. CTR).

At the end of treatment, neutral lipids were visualized by Oil Red O bright-field microscopy or BODIPY fluorescence microscopy. In cells treated with the 24 h + 24 h protocol, the red or fluorescent granules were reduced by about 10–20% compared to cells treated for 24 h but still well above the basal level (Figure 8A,B). The blow-ups of selected microscopic fields of ORO- and BODIPY-stained cells clearly show that steatosis was largely preserved under the modified protocol, as supported by quantification in the relative histograms. This sequential induction–washout design separates lipid loading from pharmacological modulation while maintaining steatosis during compound exposure.

**Figure 9:**
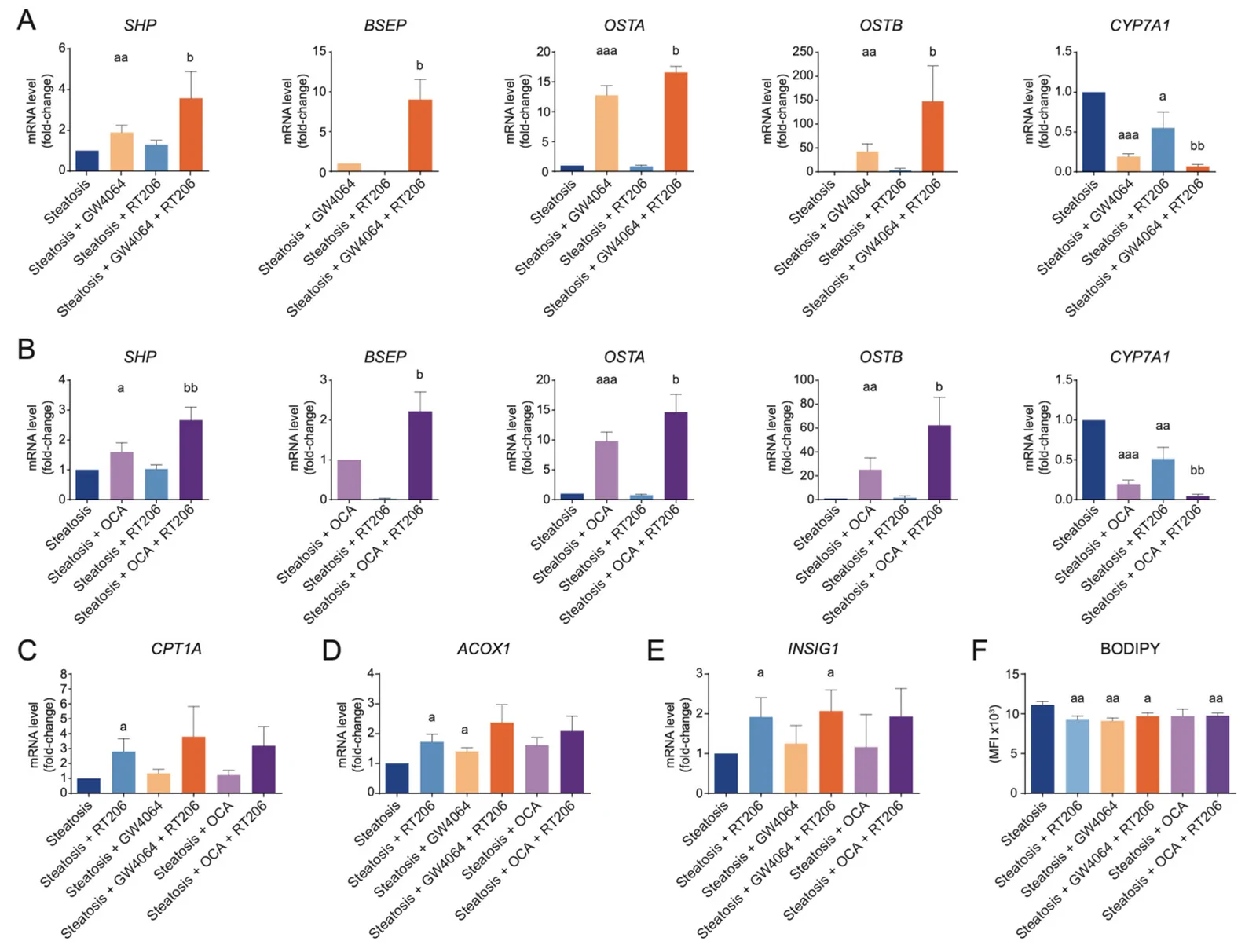
RT206 retains the ability to modulate PPAR- and FXR-dependent genes in a sequential fatty acid-loading model. (**A**) RT-qPCR analysis of *SHP* (n = 4), *BSEP* (n = 3), *OSTA* (n = 3), *OSTB* (n = 4), and *CYP7A1* (n = 3) expression in HepG2 cells exposed to the fatty acid mixture for the first 24 h and subsequently treated with GW4064 (1 µM), RT206 (20 µM), or their combination for an additional 24 h upon fatty acid and BSA removal. (**B**) RT-qPCR analysis of the same genes (*SHP* (n = 4), *BSEP* (n = 3), *OSTA* (n = 4), *OSTB* (n = 4), and *CYP7A1* (n = 3)) in cells exposed to the fatty acid mixture for the first 24 h and subsequently treated with OCA (10 µM), RT206 (20 µM), or their combination for an additional 24 h upon fatty acid and BSA removal. Because *BSEP* expression in steatotic cells was very low or undetectable, *BSEP* fold-changes in panels A and B were calculated relative to GW4064 and OCA treatments, respectively. (**C**–**E**) RT-qPCR analysis of *CPT1A* (n = 4) (**C**), *ACOX1* (n = 3) (**D**), and *INSIG1* (n = 4) (**E**) in cells treated as described in panels (**A**,**B**). (**F**) Flow-cytometric quantification of BODIPY-stained neutral lipids in cells treated as in (**A**,**B**), reported as mean fluorescence intensity in the FITC channel (n = 4). RT-qPCR data were normalized to 18S RNA. Statistical significance was considered when a *p* < 0.05, aa *p* < 0.01 or aaa *p* < 0.001 (*t*-test with Bonferroni correction vs. Steatosis); b *p* < 0.05, bb *p* < 0.01 (*t*-test vs. Steatosis + GW4064 or Steatosis + OCA).

Under the 24 h + 24 h protocol, *SHP*, *BSEP*, *OSTA* and *OSTB* were induced by GW4064 and further enhanced by the GW4064/RT206 combination, restoring the cooperative phenotype observed under basal conditions (Figure 9A). Conversely, *CYP7A1* was reduced by approximately 50% with RT206, 80% with GW4064, and nearly completely suppressed by the combination. Thus, removal of BSA during compound exposure preserved steatosis and restored cooperative transcriptional modulation.

Similar data were obtained using OCA (10 μM) instead of GW4064. *SHP*, *BSEP*, *OSTA* and *OSTB* were induced by OCA and further enhanced by OCA/RT206 co-treatment, whereas *CYP7A1* repression was strengthened under combination treatment (Figure 9B). Maintenance of cooperative enhancement with two structurally distinct orthosteric agonists shows that RT206-mediated potentiation is not restricted to a single orthosteric chemotype and is consistent with the proposed S2-binding model.

We also evaluated the expression of *CPT1A*, *ACOX1*, and *INSIG1*, PPAR-regulated genes involved in fatty acid degradation and cholesterol metabolism. The mRNA levels of all three genes were increased by RT206 alone and to a similar extent by the GW4064/RT206 combination (Figure 9C–E). Finally, we verified whether the same treatments were able to reduce lipid accumulation typical of the steatosis model. We quantified the neutral lipid content in cells under the modified protocol and upon treatment by BODIPY staining in flow cytometry analysis. Mean fluorescence intensity (MFI) showed significant lipid accumulation in steatotic cells, which was modestly reduced by RT206 (~15%) and by GW4064 or OCA (~15%), and slightly further by the combinations (Figure 9F).

Collectively, these results demonstrate that RT206 retains functional activity in a steatotic hepatocyte model, acting as a partial PPARα/γ agonist and potentiating FXR signaling in the presence of orthosteric agonists under metabolically stressed conditions. Importantly, the loss of cooperativity observed in the standard steatosis protocol was reproduced by BSA alone, consistent with reduced functional compound exposure. Recovery of the response under the 24 h + 24 h protocol supports preservation of ligand-dependent FXR potentiation after lipid loading.

## 4. Discussion

In this study, we demonstrate that RT206, a 2-aryloxy-3-phenyl-propanoic acid chemotype, acts as a partial agonist of PPARα and PPARγ and, importantly, as a positive allosteric modulator of the bile-acid receptor FXR. This dual pharmacology constitutes the main mechanistic advance of this work and supports RT206 as a proof-of-principle scaffold that links transcriptional programs associated with fatty acid oxidation to FXR-regulated bile acid metabolism [9,12].

Molecular docking and MD simulations support that RT206 occupies canonical PPARα/γ ligand-binding pockets in orientations compatible with partial agonism, and this structural hypothesis is corroborated by partial activation in GAL4-PPAR-reporter assays and induction of canonical endogenous PPAR target genes in HepG2 cells. The predicted carboxylate network of RT206 is consistent with partial stabilization of AF-2/H12, providing a structural rationale for reduced E_max_ in heterologous reporters [36]. Additional structural studies of nuclear-receptor partial agonists have shown that incomplete H12 stabilization attenuates coactivator recruitment and transcriptional efficacy, offering a mechanistic framework for the RT206 profile.

At the same time, the stronger induction of selected endogenous PPAR targets compared with reporter E_max_ is consistent with the promoter, chromatin, and co-regulator dependence of nuclear receptor outputs, which are not fully captured by simplified reporter constructs [40,59]. This gene-context dependence supports interpreting RT206 as a context-modulated partial agonist rather than a uniformly weak activator.

A key mechanistic observation is that RT206 lacks intrinsic orthosteric FXR agonism in classical transactivation assays but robustly potentiates FXR responses to full agonists (GW4064, OCA) at multiple canonical target genes, consistent with positive allosteric modulation [47,49].

GCI kinetics show weak binding to apo-FXR that is strengthened in the presence of orthosteric agonists. Together with docking and MD analyses, these data provide a coherent molecular explanation for ligand-dependent cooperativity: RT206 is predicted to occupy an S2 back-pocket whose interaction network is enhanced when S1 is liganded. Importantly, potentiation was observed with structurally distinct orthosteric agonists, showing that the effect is not restricted to a single orthosteric chemotype and supporting the proposed S2-mediated model.

FXR potentiation was not reproduced by selective PPARα or PPARγ agonists, arguing against a simple indirect mechanism based solely on PPAR-driven upregulation of FXR expression and supporting a receptor-level component. Although this control does not exclude all forms of transcriptional crosstalk, it strengthens the interpretation of ligand-dependent FXR modulation.

Combined PPAR–FXR modulation has been explored previously, but primarily through orthosteric multi-agonism, including dual FXR/PPARδ activators [67], a triple FXR/PPARα/δ agonist that reversed fibrosis in a diet-induced mouse model of NASH [68], dual FXR/PPARγ partial agonists developed to limit the excessive activation associated with full agonists [69], and benzoxazole-based FXR/PPARα dual agonists [70]. In these compounds, each receptor is activated through direct engagement of its canonical orthosteric pocket. RT206, instead, acts as an orthosteric partial agonist at PPARα/γ and as a ligand-dependent positive modulator of FXR, with the GCI and computational analyses supporting engagement of the S2 region when the orthosteric pocket is occupied by an FXR agonist. Whether this ligand-dependent mode of FXR modulation offers practical advantages over conventional orthosteric multi-agonism remains to be established.

Prior work documented the S2 pocket of FXR as a site for modulators that influence orthosteric ligand function; examples include GG and related steroidal modulators that act as selective bile-acid receptor modulators (SBARMs) or antagonists depending on context [56,57]. Our comparative biophysical and computational analyses reveal qualitative differences between RT206 and GG. Although both ligands are predicted to engage the S2 region, RT206 formed a directional polar and ionic anchoring network centered on R331 in the simulated ternary complexes, which was associated with reduced H12 mobility and a more stable agonist-like conformation. By contrast, the simulated GG complexes were dominated by hydrophobic contacts and showed orthosteric-ligand rearrangement, consistent with its dose-dependent antagonist or SBARM-like behavior. Thus, S2 occupancy alone may not define functional outcome: the chemical nature, orientation, and persistence of the predicted S2 interactions may contribute to the distinct functional profiles of GG and RT206 [51,52,54].

In the modeled ternary complexes, predicted RT206 occupancy at S2 was associated with reduced structural fluctuations near H12 and a more compact receptor conformation compatible with coactivator binding when an orthosteric agonist occupied S1. This modeled state provides a structural rationale for the observed potentiation and is consistent with the RMSF reductions and interaction fractions obtained from the MD trajectories. Conversely, GG increases local flexibility within the H1–H2 region and induces orthosteric drift, destabilizing the active conformation at higher concentrations; at lower doses, GG triggers alternative transcriptional programs (for example, AP-1-mediated *BSEP* induction). Cross-talk between AP-1 and FXR at proximal promoter elements likely underlies these gene-specific effects [40,58,59]. Our dose-dependent GG signatures, including *NQO1* and *MYC* induction at low concentrations and repression of FXR targets at higher concentrations, are consistent with this dual mechanism.

To assess whether these effects persist under lipid overload, we tested RT206 in a fatty acid-induced steatosis model [63,65]. RT206 combined with FXR agonists modulated genes of bile acid metabolism, upregulated fatty acid oxidation genes, and modestly reduced intracellular neutral lipids, in line with the receptors’ profiles characterized in this study.

We found that BSA used for fatty acid solubilization and delivery attenuated the effects of RT206 and the reference agonists, likely because albumin sequesters hydrophobic small molecules and reduces their free concentration [66]. When lipid loading and pharmacological exposure were temporally separated (24 h + 24 h protocol), RT206-mediated FXR potentiation and RT206-dependent PPAR activation re-emerged. Thus, the initial loss of cooperativity in the standard steatosis protocol is consistent with a vehicle-dependent reduction in functional compound exposure rather than with a context-specific loss of ligand-dependent FXR potentiation.

Several limitations of this study should be acknowledged. The liver-cell characterization of RT206 was conducted in HepG2 cells, whereas receptor selectivity was independently assessed using HEK293T GAL4-reporter assays and binding cooperativity was evaluated by cell-free GCI using purified FXR-LBD. These complementary platforms strengthen the receptor-level interpretation; nevertheless, receptor-specific loss-of-function experiments, such as genetic silencing of PPARα or PPARγ in HepG2 cells, were not performed and would be required to establish the individual contribution of each receptor to the endogenous transcriptional responses observed. In addition, the fatty acid-loaded HepG2 model primarily captures hepatocellular lipid accumulation and does not reproduce the systemic metabolic dysfunction, immune-cell involvement, or fibrogenic progression characteristic of MASLD. Accordingly, the present conclusions apply primarily to the steatotic hepatocyte rather than to the full complexity of the disease. The metabolic efficacy, safety margins, and PK/PD relationships of RT206 therefore remain to be established in appropriate in vivo models under physiologically relevant exposure conditions.

Moreover, although the functional, kinetic, and computational data support a cooperative mechanism consistent with RT206 engagement of the FXR S2 region, the proposed binding mode has not been confirmed by direct structural methods. Site-directed mutagenesis of the S2 residues predicted to anchor RT206, most notably R331, assessed in parallel transactivation and binding assays, provides a direct test of this model and defines the next stage of this work. Higher-resolution structural and co-regulator-focused studies will further refine the mechanistic model and guide medicinal-chemistry optimization of potency, selectivity, and pharmacokinetic properties.

## 5. Conclusions

**Figure 10:**
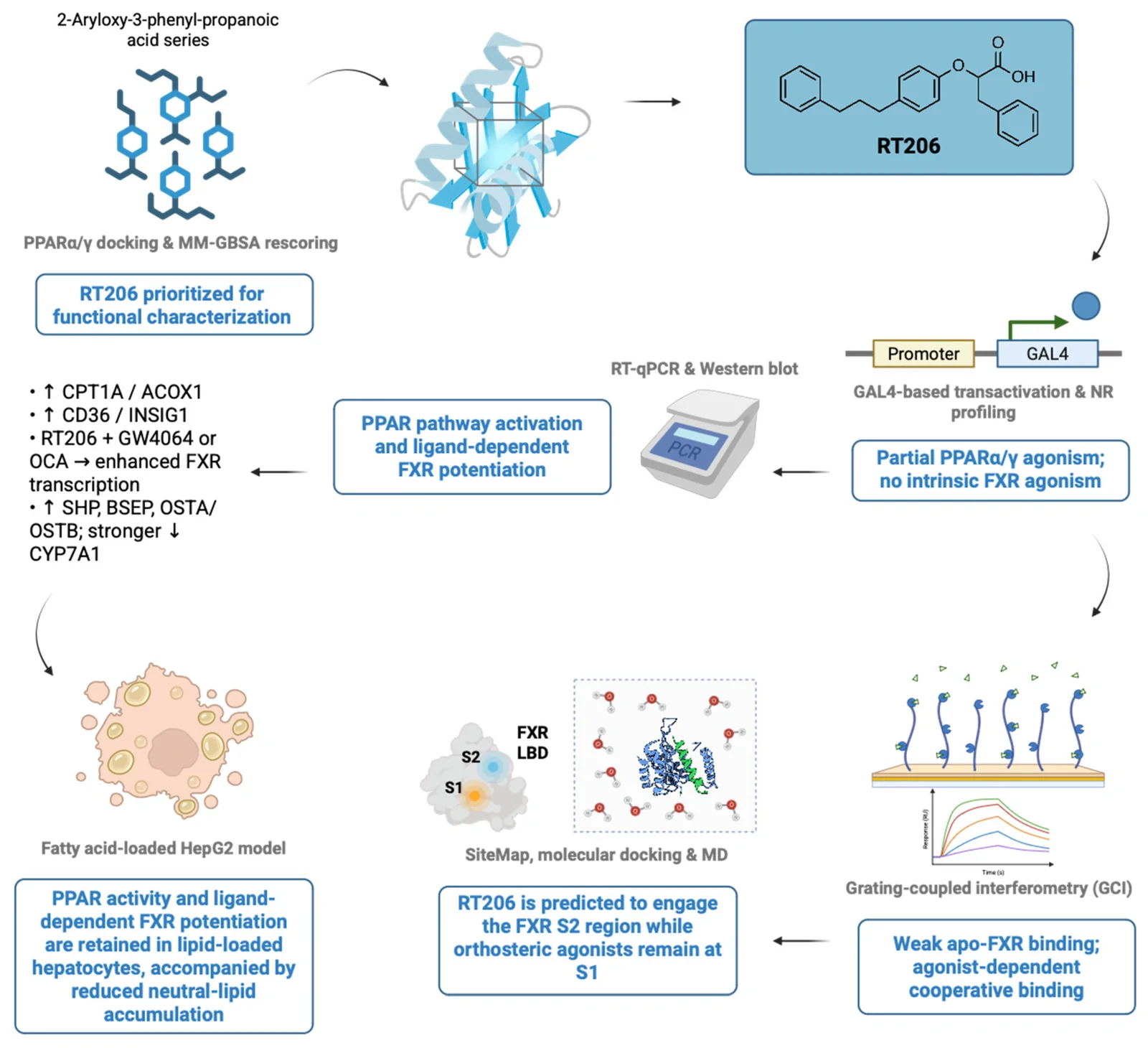
Integrated summary of the principal pharmacological and mechanistic findings for RT206. RT206 was identified as a partial agonist of PPARα and PPARγ while showing no intrinsic FXR agonist activity. In HepG2 cells, RT206 activated canonical PPAR-regulated pathways and potentiated FXR-dependent transcription in the presence of structurally distinct orthosteric agonists. GCI analyses showed weak binding to apo-FXR and agonist-dependent cooperative binding, while SiteMap analysis, molecular docking, and MD simulations supported a model in which RT206 is predicted to engage the non-orthosteric FXR S2 region while orthosteric agonists remain bound at the canonical S1 site. In fatty acid-loaded HepG2 cells, RT206 retained PPAR activity and ligand-dependent FXR potentiation, accompanied by reduced neutral-lipid accumulation. Together, these findings support an integrated pharmacological profile in which partial PPARα/γ agonism is coupled to ligand-dependent positive modulation of FXR. Arrows indicate the relationships among the principal findings and supporting experimental and computational evidence. Created in BioRender. Cerchia, C. (2026), https://BioRender.com/pdxijor.

In this study, RT206 acted as a partial PPARα/γ agonist and potentiated FXR responses to orthosteric agonists, consistent with a positive allosteric modulation profile. RT206 bound weakly to apo-FXR, whereas orthosteric agonists increased its apparent affinity. Docking and MD simulations support a model in which RT206 occupies the S2 back-pocket and forms interactions that may stabilize orthosteric-liganded FXR conformations. Comparison with GG, a modulator proposed to engage the same non-orthosteric region, revealed distinct predicted interaction patterns associated with divergent transcriptional outcomes. In fatty acid-loaded HepG2 cells, RT206 modulated FXR- and PPAR-regulated metabolic genes and modestly reduced neutral-lipid accumulation. An integrated overview of the experimental and computational workflow is provided in Figure 10.

Our data support RT206 as a proof-of-principle scaffold that combines partial PPARα/γ agonism with ligand-dependent positive modulation of FXR through a mechanism consistent with S2 engagement. This dual activity provides a chemical-biology framework for exploring the coordinated modulation of transcriptional pathways linked to fatty acid oxidation and bile acid signaling within a single molecular scaffold. Whether this pharmacological profile translates into improved efficacy or tolerability relative to existing approaches remains to be determined through dedicated in vivo and translational studies.

## Data Availability

All data generated or analyzed during this study are included in the article and its Supplementary Materials. Additional raw data are available from the corresponding authors upon reasonable request.

## Supplementary Materials

The following supporting information can be downloaded at https://www.mdpi.com/article/10.3390/biom16091316/s1, Table S1: Activity of compounds selected from the series reported by Fracchiolla et al. in cell-based PPARα and PPARγ transactivation assays, reproduced from the original publication. Table S2: MM-GBSA binding free-energy estimates calculated for selected compounds docked to PPARα and PPARγ. Table S3: SiteMap-derived properties of the additional pocket identified in different FXR ligand-binding domain structures. Table S4: Summary of the binary and ternary FXR systems investigated by molecular dynamics simulations, including starting structures, ligand-placement protocols, binding sites, and simulation sampling. Table S5: Nucleotide sequences of the primers used in this study. Table S6: Antibodies used for Western blotting in this study. Figure S1: Backbone Cα RMSD profiles of the simulated systems along three independent 1 μs molecular dynamics trajectories. Figure S2: Kinetic binding analysis of RT206 to immobilized FXR-LBD by grating coupled interferometry. Figure S3: Expression of FXR target genes and cell viability in HepG2 cells treated with OCA and RT206, alone or in combination. Figure S4: GCI kinetic analyses of GW4064 and OCA towards immobilized FXR. Figure S5: GCI signals recorded upon injection of RT206 in combination with GW4064 or OCA onto immobilized FXR. Figure S6: RT206 kinetic binding analyses in the presence of fixed concentrations of GW4064 or OCA. Figure S7: GCI kinetic analysis of GG and GG/GW4064 mixture experiments towards immobilized FXR. Figure S8: Original Western blots corresponding to Figure 1G. Figure S9: Original Western blots corresponding to Figure 1I.

## Abbreviations

The following abbreviations are used in this manuscript:

BODIPY	boron–dipyrromethene
BSA	bovine serum albumin
DMSO	dimethyl sulfoxide
FA	fatty acid
FXR	farnesoid X receptor
GCI	grating-coupled interferometry
GG	guggulsterone
LBD	ligand-binding domain
MASLD	metabolic dysfunction-associated steatotic liver disease
MD	molecular dynamics
MM-GBSA	molecular mechanics–generalized Born surface area
MFI	mean fluorescence intensity
OCA	obeticholic acid
ORO	Oil Red O
PPAR	peroxisome proliferator-activated receptor
PK/PD	pharmacokinetics/pharmacodynamics
qPCR	quantitative polymerase chain reaction
RMSD	root mean square deviation
RMSF	root mean square fluctuation
