# Supplementary material for: RT206 Couples Partial PPARα/γ Agonism with Ligand-Dependent Allosteric Potentiation of FXR Signaling in Liver Cells
Source: Biomolecules. 2026 Sep 10;16(9):1316. doi: 10.3390/biom16091316 (PMC13604256; doi:10.3390/biom16091316)
Supplement: Supplementary file 1 [file biomolecules-16-01316-s001.zip › biomolecules-4515826-supplementary.pdf]

RT206 Couples Partial PPARα/γ Agonism with Ligand-Dependent Allosteric Potentiation of FXR Signaling in Liver Cells

Manuela Leo,[1,†] Carmen Cerchia,[2,†] Antonio Laghezza,[3] Francesca Rinaldi,[4] Enrica Calleri,[4] Vittorio Colantuoni,[1] Fulvio Loiodice,[3] Lina Sabatino,[1,*] and Antonio Lavecchia[2*]

[1]Department of Sciences and Technologies, University of Sannio, 82100 Benevento, Italy

[2]Drug Discovery Laboratory, Department of Pharmacy, University of Naples "Federico II", 80131 Naples, Italy

[3]Department of Pharmacy-Drug Science, University of Bari "Aldo Moro", 70126 Bari, Italy

[4]Department of Drug Sciences, University of Pavia, 27100 Pavia, Italy

†These authors contributed equally

*Correspondence: lina.sabatino@unisannio.it (L.S.), antonio.lavecchia@unina.it (A.L.)

Supplementary Materials

Table S1. PPARα and PPARγ transactivation data for compounds selected from the series previously reported by Fracchiolla et al.

Compound	X	PPARα EC50 (μM)	PPARα Efficacy	PPARγ EC50 (μM)	PPARγ Efficacy
**S-4**	CH_2CH_2	0.15 ± 0.04	83 ± 10	0.40 ± 0.20	33 ± 5
**15**	$CH_2CH_2CH_2$	0.72 ± 0.20	67 ± 15	0.87 ± 0.16	30 ± 6
**14**	C≡C	1.36 ± 0.06	36 ± 3	0.540 ± 0.017	39 ± 1
**12**	CH=CH trans	1.75 ± 0.12	57 ± 4	0.72 ± 0.27	50 ± 1
**R-2**	CH_2	2.4 ± 0.8	82 ± 2	5.7 ± 0.4	21 ± 1
**9**	CH_2NH	3.6 ± 1.5	78 ± 4	13 ± 3	27 ± 6

Table S2. MM-GBSA binding free energy estimates calculated on docked complexes with PPARα and PPARγ.

Compound	X	MM-GBSA ΔG_{bind} (kcal mol−1) (PPARα)	MM-GBSA ΔG_{bind} (kcal mol−1) (PPARγ)
**S-4**	CH_2CH_2	-62.17	-50.94
**S-15**	$CH_2CH_2CH_2$	-65.52	-57.17
**R-15**	$CH_2CH_2CH_2$	-64.55	-51.16

Table S3. SiteMap-derived properties of the identified pocket within different FXR LBD structures. The threonine residue previously identified by mutagenesis is highlighted in bold.

PDB	Ligand	SiteScore	Size	Dscore	Volume (Å³)	Residues
6HL1	CDCA	1.097	100	1.009	150.234	Chain A: 259, 260, 261, 263, 264, 265, 293, 294, 297, 298, 301, 327, 330, 331, 334, 379, **386**
3DCT	GW4064	1.018	145	0.971	312.473	Chain A: 259, 260, 261, 262, 263, 264, 265, 266 ,267, 270, 293, 294, 297, 298, 300, 301, 304, 327, 330, 331, 334, 335, 337, 338, 340, 342, 372, 373, 375, 376, 377, 378, 379, 382, **386**
6HL0	APO	0.933	36	0.88	72.03	Chain A: 260, 263, 294, 295, 297, 298, 301, 324, 327, 328, 331, **386**, 390
1OSV	OCA (RAT)	0.994	247	1.001	594.076	Chain A: 202, 256, 257, 258, 259, 260, 261, 262, 263, 264, 267, 268, 270, 287, 291, 294, 295, 298, 301, 324, 328, 331, 332, 333, 334, 335, 336, 337, 338, 339, 340, 341, 342, 343, 345, 346, 350, 360, 363, 366, 367, 374, 375, 376, 379, **383**, 387

Table S4. Summary of the systems simulated in this study.

System	Starting structure (PDB ID)	RT206 / GG binding site	Ligand placement protocol	Sampling (replicas × length)
Ternary complexes				
FXR / GW4064 / RT206	3DCT	S2 backdoor	Glide SP (grid on SiteMap-identified S2 pocket)	3 × 1 µs
FXR / OCA / RT206	6HL1[a]	S2 backdoor	Glide SP (grid on SiteMap-identified S2 pocket)	3 × 1 µs
FXR / GW4064 / GG	6HL1[b]	S2 backdoor	Induced Fit Docking (IFD)	3 × 1 µs
FXR / OCA / GG	6HL1[a]	S2 backdoor	Induced Fit Docking (IFD)	3 × 1 µs
Binary complexes				
FXR / GW4064	3DCT	n.a.	Co-crystallized pose retained	3 × 1 µs
FXR / OCA	6HL1[a]	n.a.	Ligand modeled onto the CDCA-bound pose	3 × 1 µs
FXR / RT206 (S1)	6HL1	S1 orthosteric	Glide SP (grid on co-crystallized ligand)	3 × 1 µs
FXR / RT206 (S2)	6HL1	S2 backdoor	Glide SP (grid on SiteMap-identified S2 pocket)	3 × 1 µs
FXR apo	6HL0	n.a.	n.a.	3 × 1 µs

In each simulation, the coactivator peptide co-crystallized in the starting PDB structure was retained and solvated together with the receptor.

[a] *The OCA model was generated from the CDCA-bound 6HL1 structure by modifying the co-crystallized CDCA coordinates to introduce the 6α-ethyl substituent, followed by local minimization.*
[b] *GW4064 coordinates were transferred from PDB 3DCT to 6HL1 by structural superposition of the two LBDs prior to IFD of GG into the S2 pocket.*

Table S5. Nucleotide sequences of the primers used in this study.

Target gene		Sequence	Tm (°C)
PPARG	FW	5’-CGTGGCCGCAGATTTGAA-3’	58
	RV	5’-CTTCCATTACGGAGAGATCCAC-3’	
CD36	FW	5’-CTTATACGTACAGAGTTCGTT-3’	60
	RV	5’-AGATGGCACCATTGGGCTGC-3’	
INSIG1	FW	5’-CCTTTGGTGGACATTTGATCGT-3’	60
	RV	5’-GCGTAGCTAGAAAAGCTATGGT-3’	
PPARA	FW	5’-CTATCATTTGCTGTGGAGATCG-3’	60
	RV	5’-AAGATATCGTCCGGGTGGTT-3’	
CPT1A	FW	5’-CCAGACGAAGAACGTGGTCA-3’	58
	RV	5’-ATCTTGCCGTGCTCAGTGAA-3’	
ACOX1	FW	5’-ATGGTGTTTGTCAGGTCCTTCCT-3’	62
	RV	5’-CTGGGTTTGAAAATCCAAAATCT-3’	
CYP7A1	FW	5’-CACAATGCCCGGGAGAAA-3’	56

	RV	5’-AAAGTCGCTGGAATGGTGTTTG-3’	
SHP	FW	5’-CTCTTCCTGCTTGGGTTGGC-3’	60
	RV	5’-GCACATCGGGGTTGAAGAGG-3’	
BSEP	FW	5’-ACATGCTTGCGAGGACCTTTA-3’	60
	RV	5’-GGAGGTTCGTGCACCAGGTA-3’	
OSTA	FW	5’-TTCCTCTAAAACCAGGTCTCAAGT-3’	62
	RV	5’-GCACAGTCATTAGAAAAGTCTCCA-3’	
OSTB	FW	5’-CAGGAGGTGCTGGAAGAGAT-3’	62
	RV	5’-GACCATGCTTATAATGACCACCA-3’	
NQO1	FW	5’-GAAGAGCACTGATCGTACTGGC-3’	60
	RV	5’-GGGTCCTTCAGTTTACCTGTGAT-3’	
MYC	FW	5’-CACCAGCAGCGACTCTGA-3’	60
	RV	5’-GATCCAGACTCTGACCTTTTGC-3’	
18S	FW	5’-GGGAGCCTGAGAAACGGC-3’	58-60
	RV	5’-GGGTCGGGAGTGGGTAATTT-3’	

Table S6. Antibodies used for Western blotting in this study.

**Target protein**	**Reference**	**Host**	**Supplier**	**Dilution**
Anti-PPARγ	2443	Rabbit	Cell Signaling Technologies	1:1000
Anti-PPARα	ab227074	Rabbit	Abcam	1:1000
Anti-α-Tubulin	T5168	Mouse	Merck	1:10000
Anti-Rabbit-HRP	7074	-	Cell Signaling Technologies	1:2000
Anti-Mouse-HRP	7076	-	Cell Signaling Technologies	1:2000

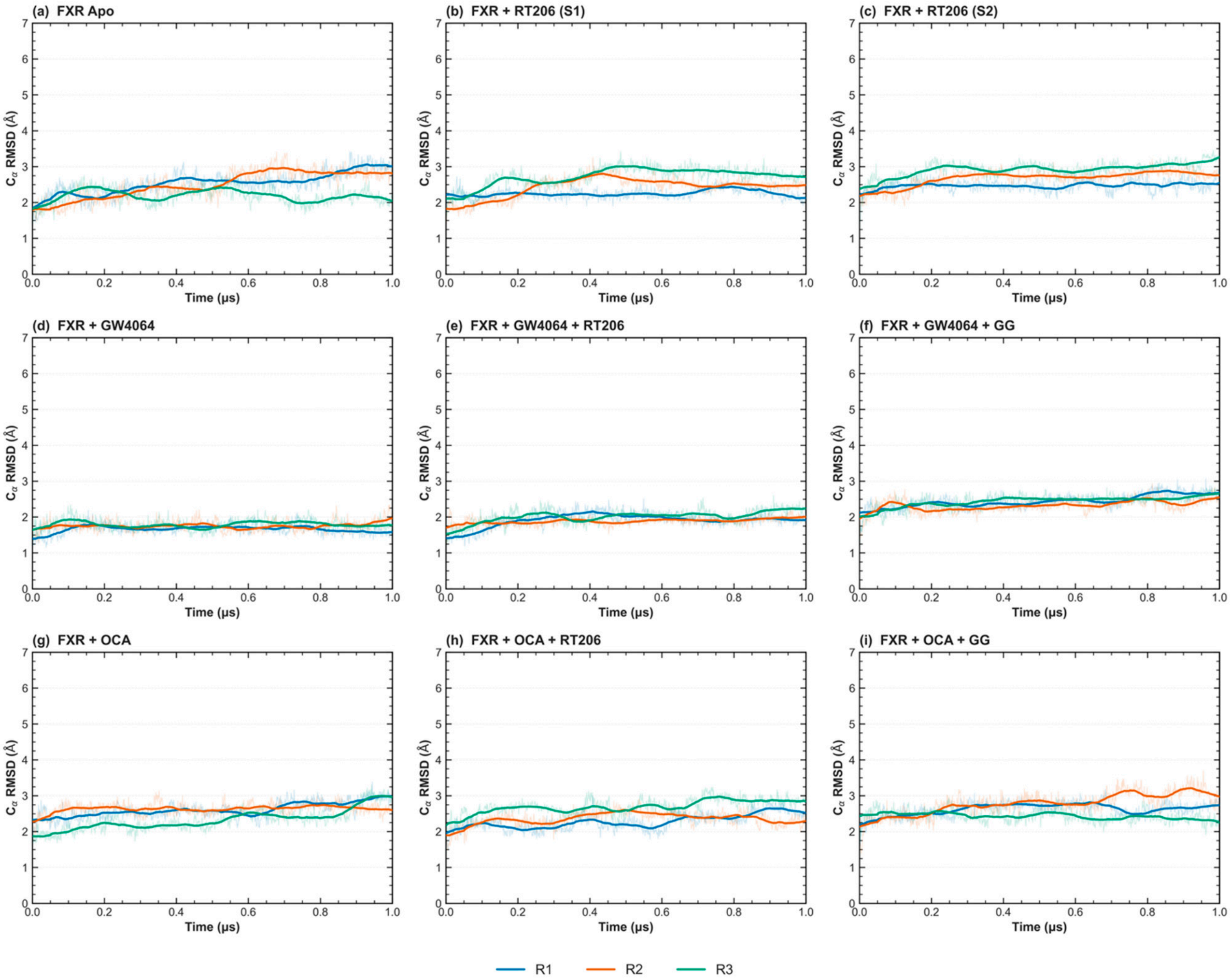

Figure S1. Backbone Cα RMSD profiles of the simulated systems along 1 μs MD trajectories. **A)** apo-FXR; **B)** FXR + RT206 docked in the orthosteric S1 pocket; **C)** FXR + RT206 docked in the S2 backdoor pocket; **D)** FXR + GW4064; **E)** FXR + GW4064 + RT206; **F)** FXR + GW4064 + GG; **G)** FXR + OCA; **H)** FXR + OCA + RT206; **I)** FXR + OCA + GG. For each system, three independent replicas (R1, blue; R2, orange; R3, green) are shown; light traces correspond to the raw frame-wise RMSD values, while solid bold traces represent the moving average (window = 50 ns) and are provided to aid visual comparison across systems.

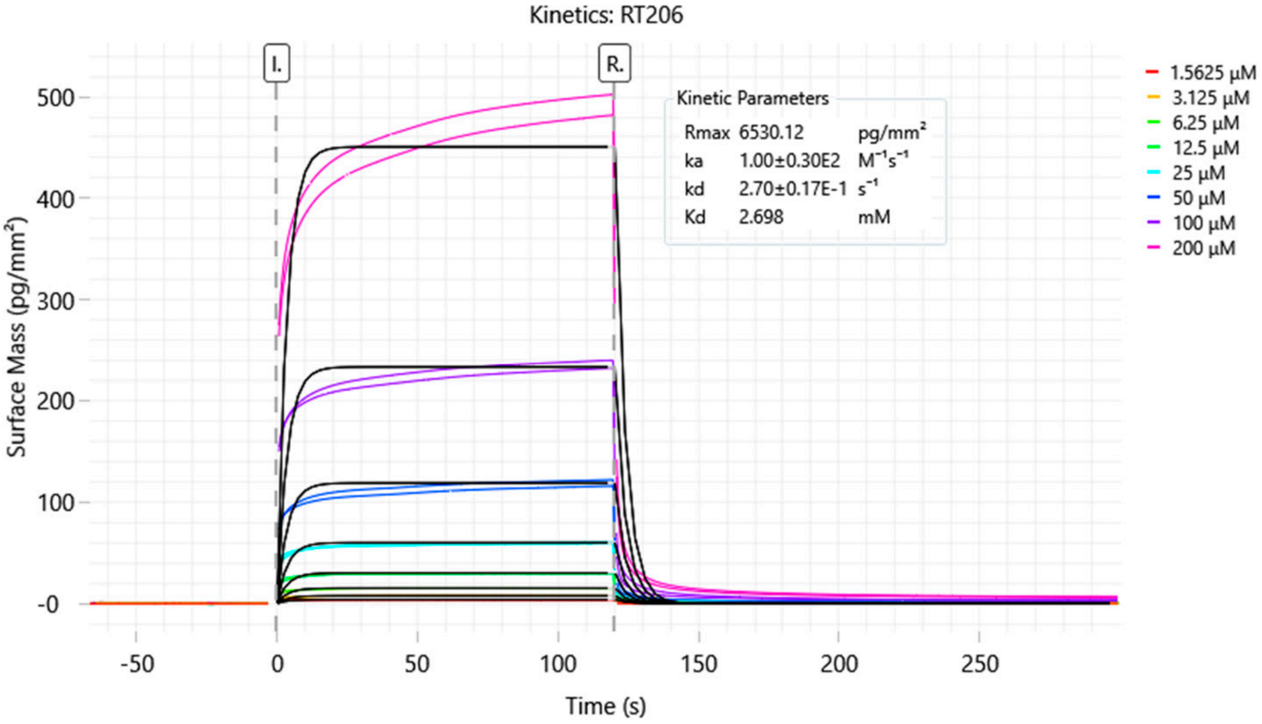

Figure S2. Kinetic binding analysis of RT206 to immobilized FXR-LBD by grating-coupled interferometry.

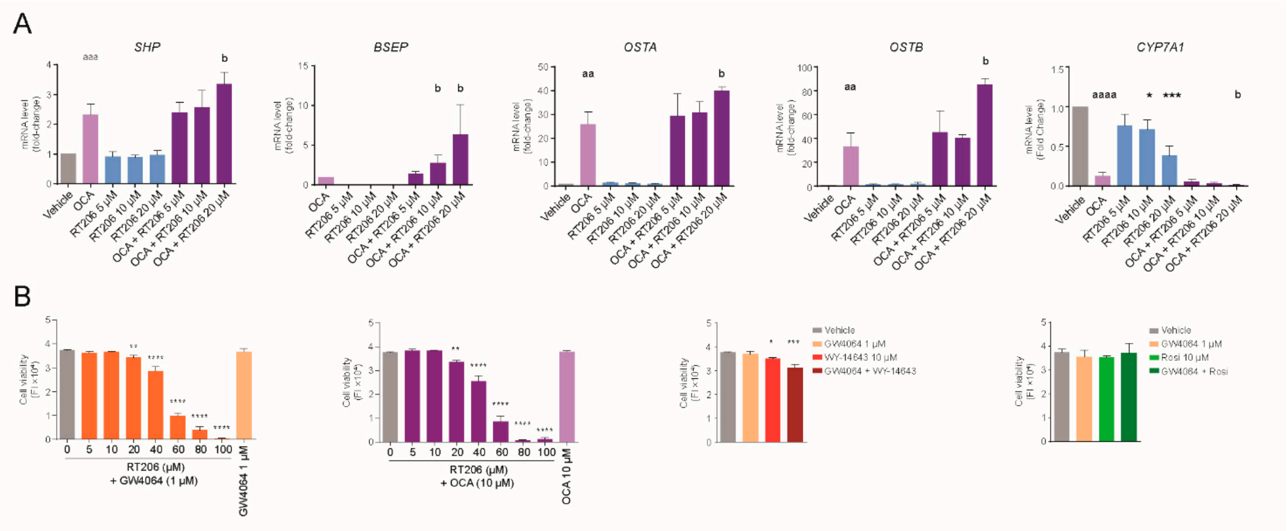

Figure S3. A) Expression of *SHP* (n=4), *BSEP* (n=5), *OSTA* (n=3), *OSTB* (n=3), and *CYP7A1* (n=3) genes in HepG2 cells treated for 24 h with the vehicle (DMSO) or OCA (10 μM), the indicated doses of RT206, alone or in combination. **B)** Viability (n=3) of HepG2 cells treated for 24 h with the indicated concentrations of RT206 alone or in combination with GW4064, OCA, WY-14643, or rosiglitazone. All histograms report normalization of mRNA levels to 18S in RT-qPCR analysis. Only for *BSEP* expression, the fold-change is referred to OCA treatment, as the basal level is very low or undetermined. Statistical significance was considered when a $p < 0.05$, aa $p < 0.01$, aaa $p < 0.001$, or aaaa $p < 0.0001$ (t-test *vs* Vehicle); b $p < 0.05$ (t-test with Bonferroni correction *vs* OCA); $*$ $p < 0.05$, $**$ $p < 0.01$, $***$ $p < 0.001$, or $****$ $p < 0.0001$ (ANOVA with Dunnett's post-test *vs* Vehicle).

A

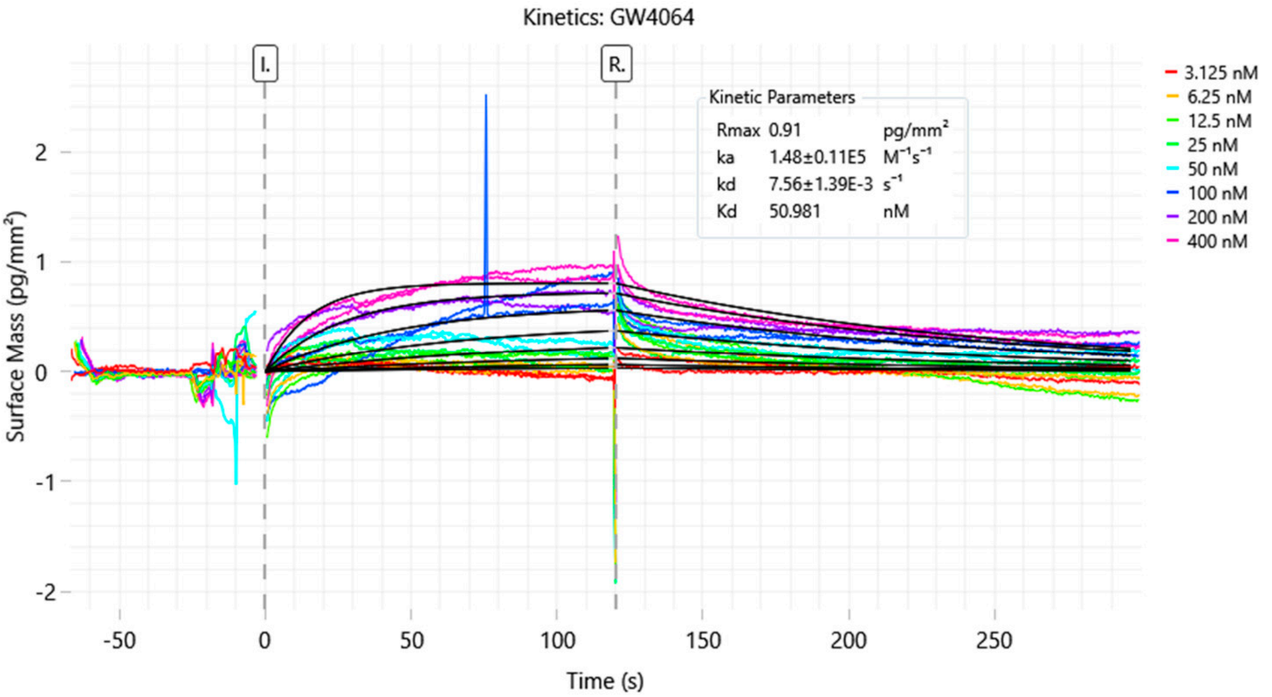

B

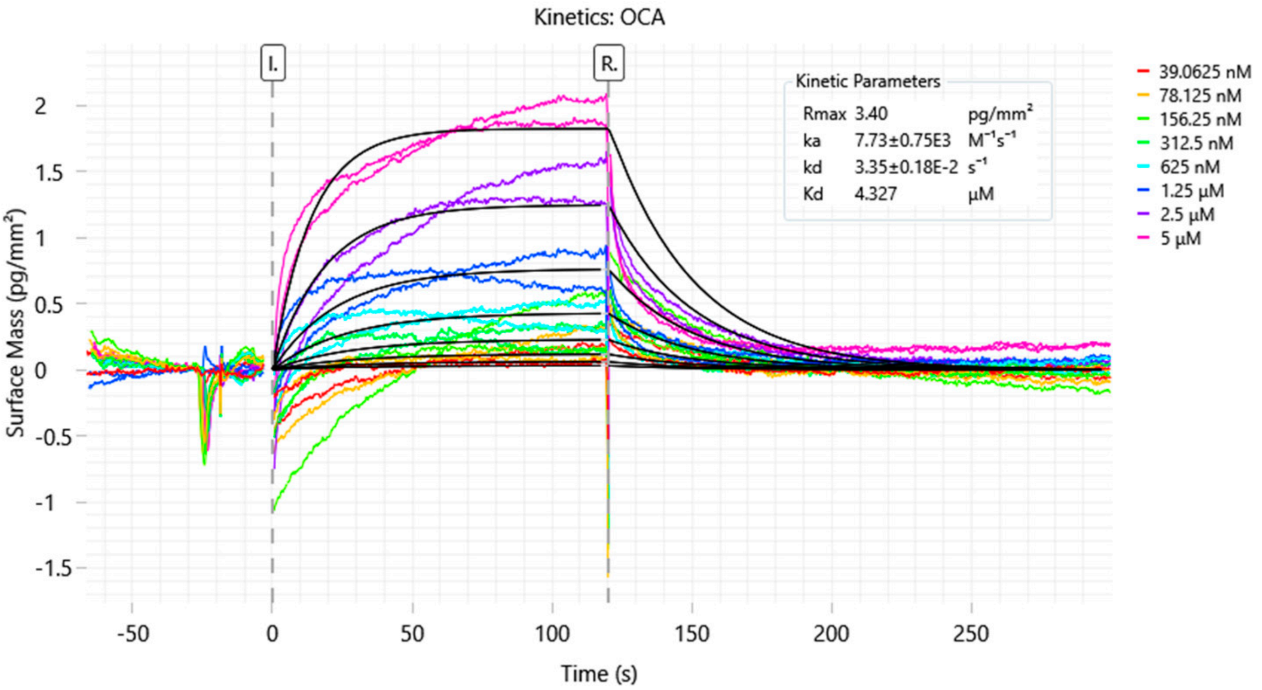

Figure S4. A) GW4064 GCI kinetic analysis towards immobilized FXR; **B)** OCA GCI kinetic analysis towards immobilized FXR.

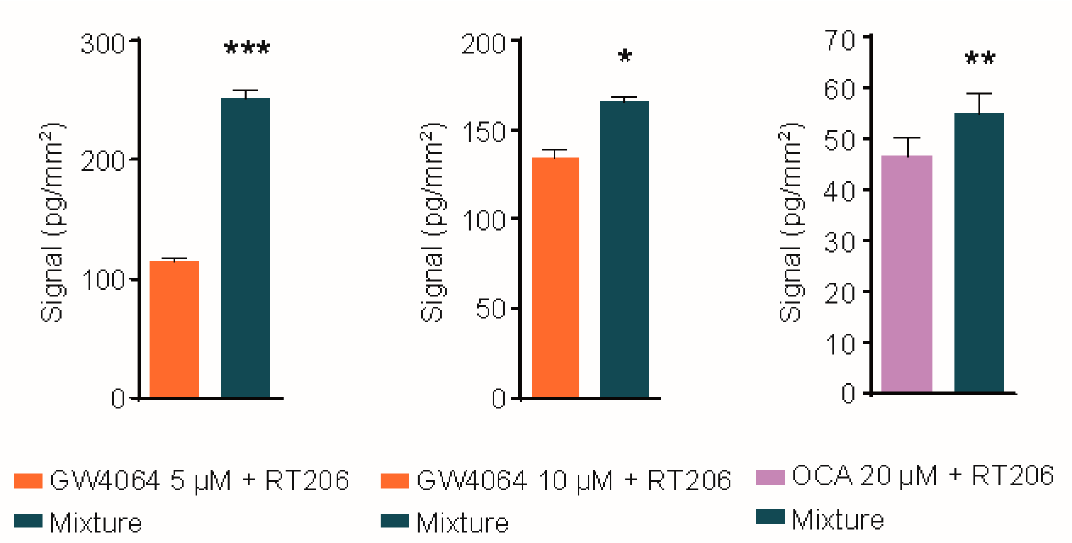

Figure S5. GCI signals recorded upon injection of 100 µM RT206 and 5 or 10 µM GW4064 either individually (orange bar) or in combination (blue bar) onto immobilized FXR. The same experiment was conducted by injecting 100 µM RT206 and 20 µM OCA either individually (violet bar) or in combination (blue bar). Statistical significance was considered when $*p < 0.05$, $**p < 0.01$, and $***p < 0.001$ (paired t-test).

A

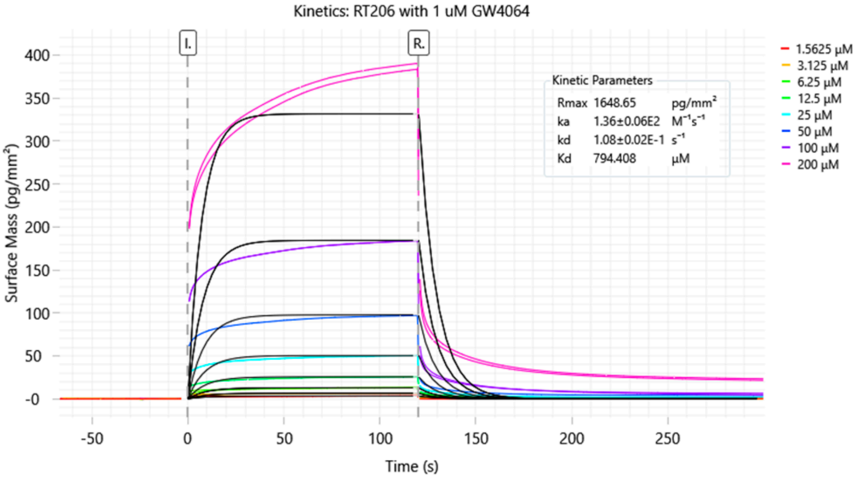

B

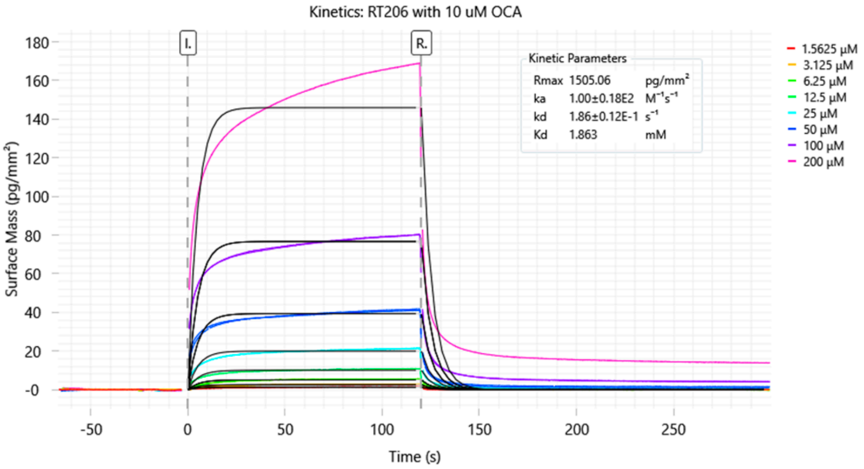

C

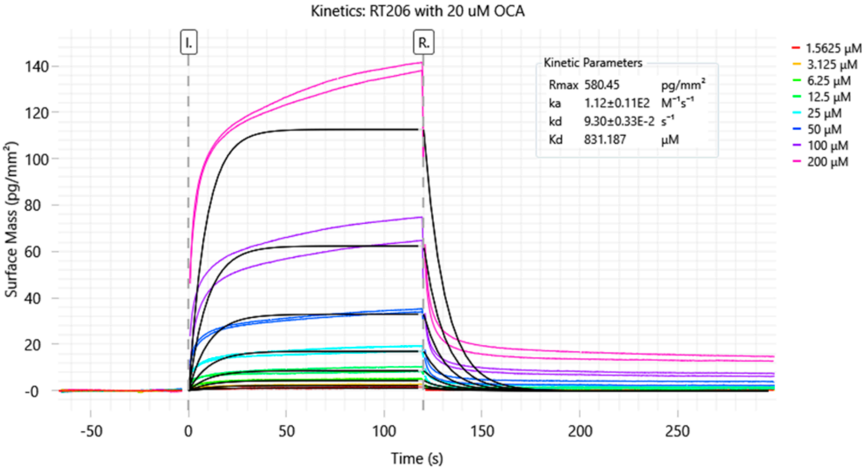

Figure S6. RT206 kinetic analysis of binding to immobilized FXR in presence of a fixed concentration of **A)** GW4064 1 µM or **B)** OCA 10 µM or **C)** OCA 20 µM.

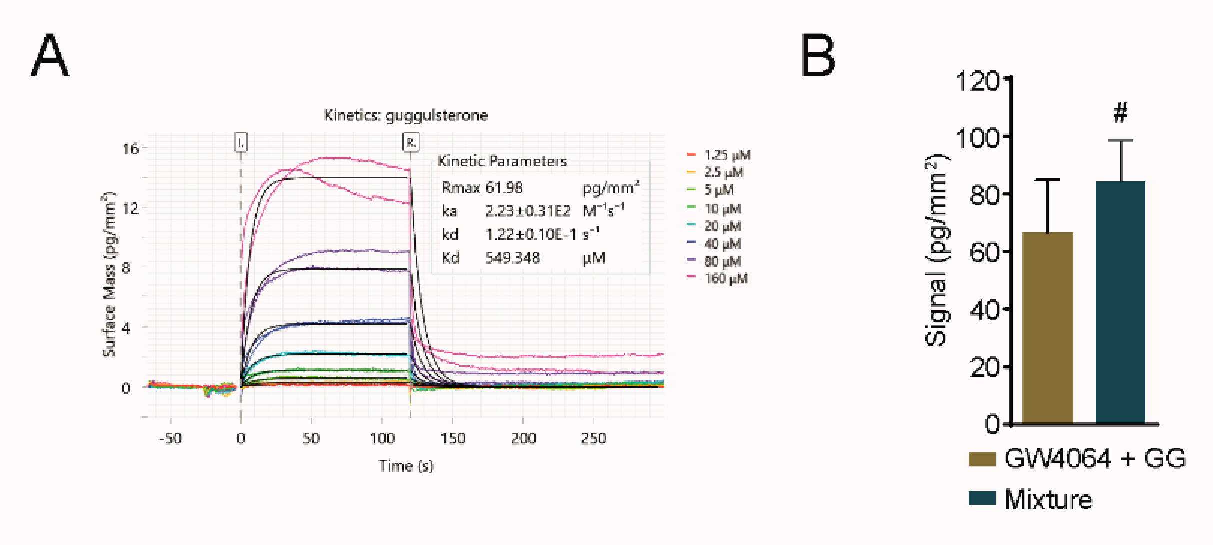

Figure S7. A) GG kinetic analysis towards immobilized FXR; **B)** GCI signals recorded upon injection of 10 µM GW4064 and 100 µM GG either individually (brown bar) or in combination (blue bar) onto immobilized FXR. Statistical significance was considered when # $p < 0.05$ (paired t-test).

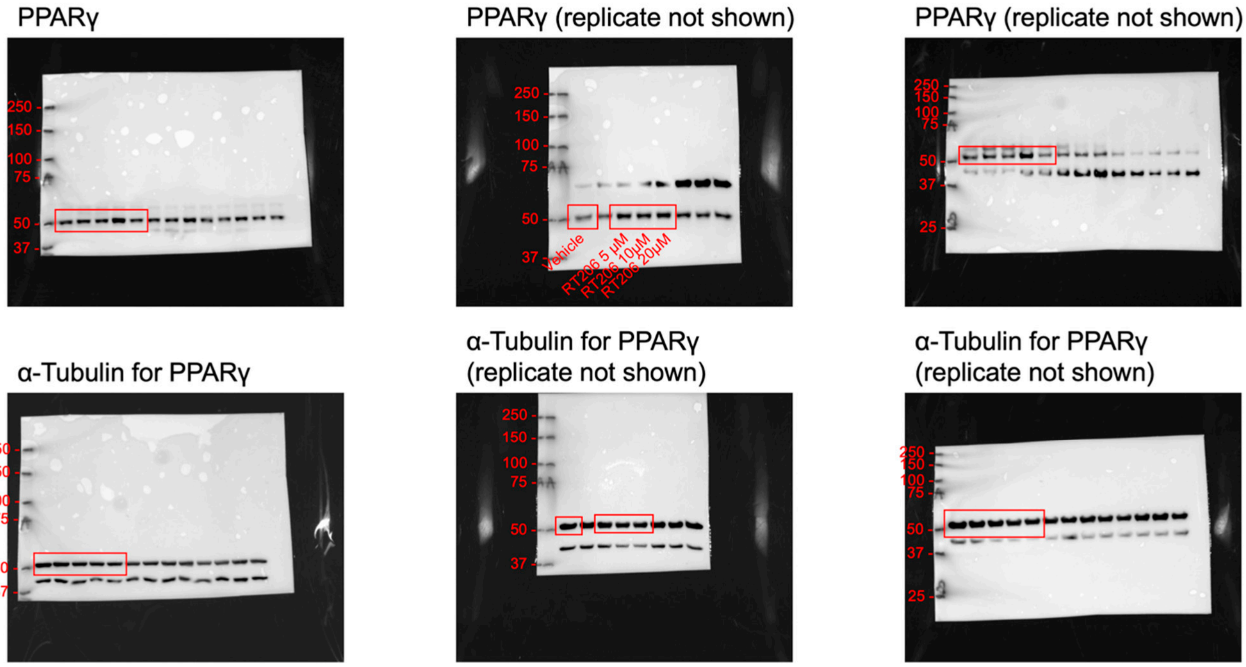

Figure S8. Original Western blots corresponding to Figure 1G. Uncropped Western blot images showing detection of PPARγ in extracts of HepG2 cells treated as indicated in Figure 1G legend. The PPARγ bands, circled in red in the upper panels, are from three independent experiments. Band density was normalized to α-Tubulin bands (outlined in red in the lower panels) from the same blots, using ImageLab software, version 6.1.0.

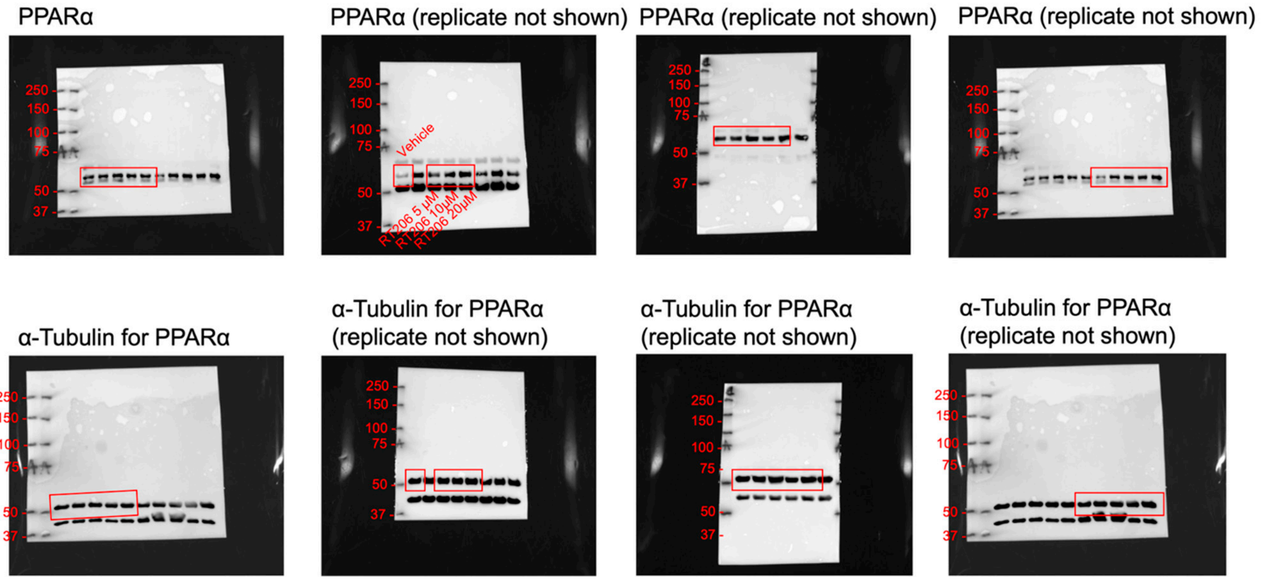

Figure S9. Original Western blots corresponding to Figure 1I. Uncropped Western blot images showing detection of PPARα in extracts of HepG2 cells treated as indicated in Figure 1I legend. The PPARα bands, circled in red in the upper panels, are from four independent experiments. Band density was normalized to α-Tubulin bands (outlined in red in the lower panels) from the same blots, using ImageLab software, version 6.1.0.
